# miR-9-5p in Nephrectomy Specimens is a Potential Predictor of Primary Resistance to First-Line Treatment with Tyrosine Kinase Inhibitors in Patients with Metastatic Renal Cell Carcinoma

**DOI:** 10.3390/cancers10090321

**Published:** 2018-09-10

**Authors:** Bernhard Ralla, Jonas Busch, Anne Flörcken, Jörg Westermann, Zhongwei Zhao, Ergin Kilic, Sabine Weickmann, Monika Jung, Annika Fendler, Klaus Jung

**Affiliations:** 1 Department of Urology, Charité University Medicine Berlin, 10117 Berlin, Germany; bernhard.ralla@charite.de (B.R.); jonas.busch@charite.de (J.B.); zhongwei.zhao@charite.de (Z.Z.); sabine.weickmann@charite.de (S.W.); monika.jung@charite.de (M.J.); annika.fendler@mdc-berlin.de (A.F.); 2 Department of Hematology, Oncology and Tumor Immunology, Charité University Medicine Berlin, 10117 Berlin, Germany; anne.floercken@charite.de (A.F.); joerg.westermann@charite.de (J.W.); 3 Department of Pathology, Charité University Medicine Berlin, 10117 Berlin, Germany; e.kilic@pathologie-leverkusen.de; 4 Department of Signal Transduction, Invasion and Metastasis of Epithelial Cells, Max-Delbrueck-Center for Molecular Medicine in the Helmholtz Association, 13125 Berlin, Germany; 5 Berlin Institute for Urologic Research, 10115 Berlin, Germany

**Keywords:** renal cell carcinoma, metastasis, tyrosine kinase inhibitors, sunitinib, primary resistance, microRNAs, prognostic indicator

## Abstract

Approximately 20–30% of patients with metastatic renal cell carcinoma (mRCC) in first-line treatment with tyrosine kinase inhibitors (TKIs) do not respond due to primary resistance to this drug. At present, suitable robust biomarkers for prediction of a response are not available. Therefore, the aim of this study was to evaluate a panel of microRNAs (miRNAs) in nephrectomy specimens for use as predictive biomarkers for TKI resistance. Archived formalin-fixed, paraffin embedded nephrectomy samples from 60 mRCC patients treated with first-line TKIs (sunitinib, *n* = 51; pazopanib, *n* = 6; sorafenib, *n* = 3) were categorized into responders and non-responders. Using the standard Response Evaluation Criteria in Solid Tumors, patients with progressive disease within 3 months after the start of treatment with TKI were considered as non-responders and those patients with stable disease and complete or partial response under the TKI treatment for at least 6 months as responders. Based on a miRNA microarray expression profile in the two stratified groups of patients, seven differentially expressed miRNAs were validated using droplet digital reverse-transcription quantitative real-time polymerase chain reaction (RT-qPCR) assays in the two groups. Receiver operating characteristic curve analysis and binary logistic regression of response prediction were performed. MiR-9-5p and miR-489-3p were able to discriminate between the two groups. MiR-9-5p, as the most significant miRNA, improved the correct prediction of primary resistance against TKIs in comparison to that of conventional clinicopathological variables. The results of the decision curve analyses, Kaplan-Meier analyses and Cox regression analyses confirmed the potential of miR-9-5p in the prediction of response to TKIs and the prediction of progression-free survival after the initiation of TKI treatment.

## 1. Introduction

The introduction of targeted therapy agents has significantly improved the overall survival of patients with metastatic renal cell carcinoma (mRCC) [1]. The tyrosine kinase inhibitors (TKIs) sunitinib, pazopanib or sorafenib are frequently used standard-of-care options for the first-line treatment of these patients [2,3]. The tumor response to these drugs is generally evaluated according to the standard Response Evaluation Criteria in Solid Tumors (RECIST) based on computed tomographic or magnetic resonance imaging data of the metastatic lesions [4,5]. A recent large sunitinib global expanded-access trial with 3353 evaluated mRCC patients reported an objective response rate of approximately 20% (defined as the sum of complete and partial responses according to RECIST criteria) and a clinical benefit (sum of objective response plus stable disease ≥3 months) of approximately about 76% [6]. Thus, 20–30% of patients are primarily refractory to sunitinib treatment. This phenomenon of non-responsiveness is termed primary or intrinsic resistance, in contrast to the secondary or acquired resistance that often develops after 6–15 months of treatment [7]. In both cases, the mechanisms are obviously multifactorial and have not yet been satisfactorily examined [8,9].

A reliable prediction of primary resistance to TKIs during first-line therapy could be helpful to avoid unnecessary treatment of patients and to save time in selecting other potentially beneficial therapeutic options [2]. Since patients with a primary resistance exhibit a poor overall survival [10,11], they could also be classified by a successful prediction of this resistance into a different prognostic group according to different clinical score systems before initiation of first-line targeted therapy [12,13,14]. Current prognostic measures consist of hematologic values, a clinical performance index and the time interval between first RCC diagnosis and occurrence of metastases (mostly after partial nephrectomy), but their capability is limited to correctly predicting the response to targeted therapy [15].

Thus, a necessary research task consists of the identification of new molecular markers that could, alone or in combination with the conventional clinicopathological variables, improve reliable prediction of therapeutic responses for mRCC patients [15,16]. The expression of several tissue and circulating analytes has been shown to correlate with the efficacy of sunitinib or other targeted agents in mRCC patients (reviewed in [9,17,18]). However, so far, there has been no approved biomarker to correctly predict the response to targeted therapy in these patients [17,19]. In this regard, microRNAs (miRNAs, miRs) as small, non-protein-coding transcripts could be considered as suitable biomarkers because of their important role as posttranscriptional regulators in all network processes of carcinogenesis and metastasis [20]. Several studies that have identified differentially expressed miRNAs in nephrectomy specimens of RCC patients with subsequent tumor relapse while under TKI therapy have confirmed this assumption [21,22,23,24,25,26,27]. However, different definitions of the clinical endpoints of progression-free survival or time to progression were used in these studies to classify two groups with different responses to sunitinib or other TKIs. As clearly stated in a currently published review of the studies carried out so far, this is, among other reasons, one decisive cause for the partly inconsistent results between the studies [28]. Therefore, we used a more practical outcome scenario. We defined patients as non-responders having primary resistance if at the first RECIST re-assessment (generally within 3 months) after the start of treatment with TKI they were found to have progressive disease and we defined patients as responders if they had complete or partial response and stable disease under TKI for at least 6 months.

Under these conditions, the aims of this study were (a) to identify typical miRNA profiles in nephrectomy specimens from two groups of mRCC patients under first-line TKI treatment, (b) to explore the most discriminative miRNAs as predictive biomarkers of primary resistance to first-line TKI therapy by reverse-transcription quantitative real-time polymerase chain reaction (RT-qPCR) using the sophisticated droplet digital PCR technique and (c) to validate the predictive capability using different evaluation approaches such as receiver-operating characteristic curve analysis (ROC), decision curve analysis and univariate and multivariate analyses in comparison to that for conventional clinicopathological variables. Collectively, this study was primarily focused on the possible development of a potential predictive marker of primary resistance in the first-line treatment of mRCC patients with TKIs.

## 2. Materials and Methods

### 2.1. Patients and Samples

**Figure 1:**
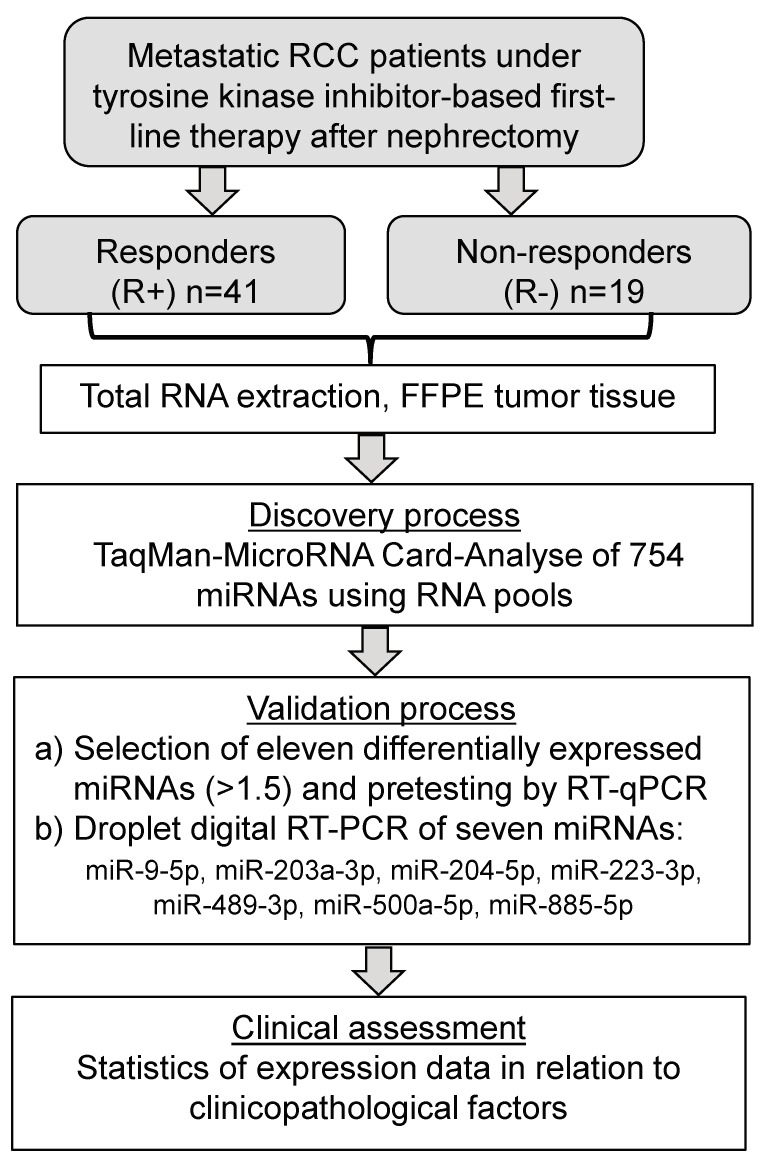
Flowchart of the study. Abbreviations: FFPE, formalin-fixed, paraffin-embedded; RCC, renal cell carcinoma; RT-qPCR, reverse transcription real time quantitative polymerase chain reaction; TKIs, tyrosine kinase inhibitors.

The study was approved by the local Ethics committee of the University Hospital Charité (EA1/153/07; EA1/134/12) and informed patient consent was obtained. The study was carried out in accordance with the Declaration of Helsinki. The general criteria of the guidelines “Standards for Reporting of Diagnostic Accuracy” (STARD) and “Reporting Recommendations for Tumor Marker Prognostic Studies” (REMARK) were considered [29,30]. The flow diagram shown in Figure 1 outlines the steps performed in this retrospective study completed in July 2017.

The study included 60 RCC patients who underwent radical nephrectomy from 2000 through 2011 and were subsequently treated with TKIs as first-line therapy using the standard treatment schedule after they developed metastasis (Table 1). The patients had not previously received other therapies. Criteria of the RECIST guidelines were used to characterize the response to this treatment [4,5]. According to these criteria, after the first restaging assessment that was generally performed 3 months after the initiation of TKI treatment, patients with progressive disease were considered as non-responders with primary resistance and those patients with partial response or stable disease under the TKI treatment for at least 6 months as responders.

Tumors were classified according to the 2002 TNM classification and the Fuhrman grading system by an experienced pathologist (E.K.) [31,32]. Archived FFPE tissue samples that were prepared from the surgically removed tumors for routine diagnostic purposes were used in this study. Hematoxylin-eosin stained histological FFPE sections were prepared to identify areas of normal and tumor tissue. These regions of interest were punch biopsied using 1.5 mm diameter needles. Only tumor samples with more than 80% cancer cells were considered for further analysis. Non-malignant tissue with a distance of >20 mm to the cancer tissue was collected from 45 patients of the study cohort for comparison purposes. These samples were selected to exclude, as far as possible, any alteration of the non-neoplastic tissue by the tumor [33,34].

### 2.2. RNA Extraction, MicroRNA Array Cards and miRNA Quantification

Total RNA was isolated from the dissected FFPE tissue samples (mean ± SD, 15.5 ± 4.6 mg) using an miRNeasy FFPE Kit (Qiagen, Hilden, Germany; Cat. No. 217504) according to the manufacturer’s instructions. Briefly, this process included steps for paraffin removal, lysis (TissueLyser, Qiagen; 2 × 1 min, 30 Hz, 5 mm stainless steel beads) with proteinase K digestion at 50 °C, on-column DNase I digestion, binding and washing of total RNA including miRNA on a MinElut spin column membrane. Total RNA was eluted from the membrane with 16 µL of nuclease-free water and the RNA concentration and purity were measured on a NanoDrop ND-1000 spectrophotometer (NanoDrop Technologies, Wilmington, DE, USA). The isolated RNA was stored at −80 °C until analysis.

For miRNA signature profiling, the preconfigured TaqMan^®^ Array Human MicroRNA A + B Cards Set v3.0 (Thermo Fisher Scientific, Waltham, MA, USA; Cat. No. 4444913) was applied according to the manufacturer’s instructions. Two RNA sample pools consisting of equal RNA aliquots from ten different samples of the patients of each group were used for multiplexed cDNA synthesis. cDNAs were measured on a ViiA7 real-time PCR system (Thermo Fisher Scientific) under default thermal-cycling conditions for the 384-well TaqMan microRNA array cards. Quantification cycles (Cq values) were generated by SDS software v2.3 and were exported for further calculations. Further details are given in Supplementary Information S1 and Supplementary Table S1.

For the validation of miRNA profiling results, a two-step procedure was chosen. RT-qPCR measurements were performed first on a Light-Cycler 480 (Roche, Mannheim, Germany) using TaqMan miRNA assays (Thermo Fisher Scientific) as previously described [35,36,37,38] and here summarized in Supplementary Information S2 including checklist data (Supplementary Tables S2 and S3) of the MIQE guidelines “Minimum Information for Publication of Quantitative Real-Time PCR Experiments” [39] and “Minimum Information for Publication of Quantitative Digital PCR Experiments” [40] with additional Supplementary Figures S1–S3 and Supplementary Tables S4–S8. Based on the preliminary results, the validation was performed in a second step analyzing cDNAs with the droplet digital PCR technique on a droplet QX200 digital PCR instrument (Bio-Rad Laboratories GmbH, Munich, Germany). The same TaqMan assays as used on the LightCycler were utilized for the droplet digital PCR (Supplementary Table S4). An aliquot of 20 µL of the PCR mix including 10 µL of 2 × PCR Supermix for Probes (No dUTP; Cat. No. 186-3023), 1 µL of TaqMan MicroRNA Assay primer (Thermo Fisher Scientific; part No. 4427975), an miRNA-specific cDNA sample (2 µL for miR-9-5p and miR-489-3p, 1 µL of cDNA for miR-223-3p) and 7.0 or 8.0 µL of nuclease-free water was filled into one sample well and 70 µL of Droplet Generator Oil for Probes (Cat. No. 186-3005) was filled into the corresponding oil well of a DG8 Cartridge (Bio-Rad). The cartridges were closed with DG8 gaskets (Cat. No. 186-3009) and placed into the QX200 droplet generator for the partition of each sample into 20000 droplets. After droplet formation, 40 µL was transferred into a 96-well PCR plate (TwinTec PCR Plate, semi-skirted, Cat. No. 0030128575; Eppendorf, Germany). The plate was heat sealed (Pierceable Foil Heat Seal, Cat. No. 181-4040, Bio-Rad) with a PX1 PCR Plate Sealer and then placed into a PCR thermal cycler (T100 Thermal cycler, Bio-Rad). The PCR run conditions were set as follows: 10 min at 95 °C for enzyme activation, followed by 45 amplification cycles: 94 °C denaturation for 30 s and 56 °C annealing/extension for 1 min, a finishing step of enzyme deactivation at 98 °C for 10 min and a hold cooling step at 12 °C. All ramp rates were set to 2.5 °C/s. After finishing the PCR reaction, the plate was placed into a QX200 Droplet Reader and the positive/negative droplets were analyzed and counted using QuantaSoft Software v.1.7 (Bio-Rad Laboratories, Inc., CA, USA). Results are given as copies per µL of the PCR reaction mix. Further details are summarized in the checklist of the digital MIQE guidelines [40] and Supplementary Information S2 (Supplementary Figures S3 and S4, Tables S6–S8).

### 2.3. Data Analysis and Statistics

qBasePLUS, v.3.0 software (Biogazelle, Zwijnaarde, Belgium) was used for the analysis of RT-qPCR data. Statistical analyses (Mann-Whitney *U* test, Spearman rank and Pearson correlations as well as ROC, Kaplan-Meier, binary logistic regression and Cox regression analyses) were performed with SPSS 23 (SPSS Inc., Chicago, IL, USA) including the bootstrap module, GraphPad Prism 7.04 (GraphPad Software, La Jolla, CA, USA) and MedCalc 18.5 (MedCalc Statistical Software, Ostend, Belgium). The decision curve analysis [41] for models with the corresponding indicated markers were constructed as previously described [42]. A *p* value less than 0.05 (two-sided) was considered statistically significant. MedCalc was used for sample size determinations. MiRTarBase (http://miRTarBase.mbc.nctu.edu.tw/) [43], miRWalk2.0 (http://zmf.umm.uni-heidelberg.de/mirwalk2) [44] and DIANA-TarBase v7.0 (http://www.microrna.gr/tarbase) [45] databases were used to evaluate validated and predicted miRNA-target interactions.

## 3. Results

### 3.1. Study Design, Patient and Sample Characteristics

**Figure 2:**
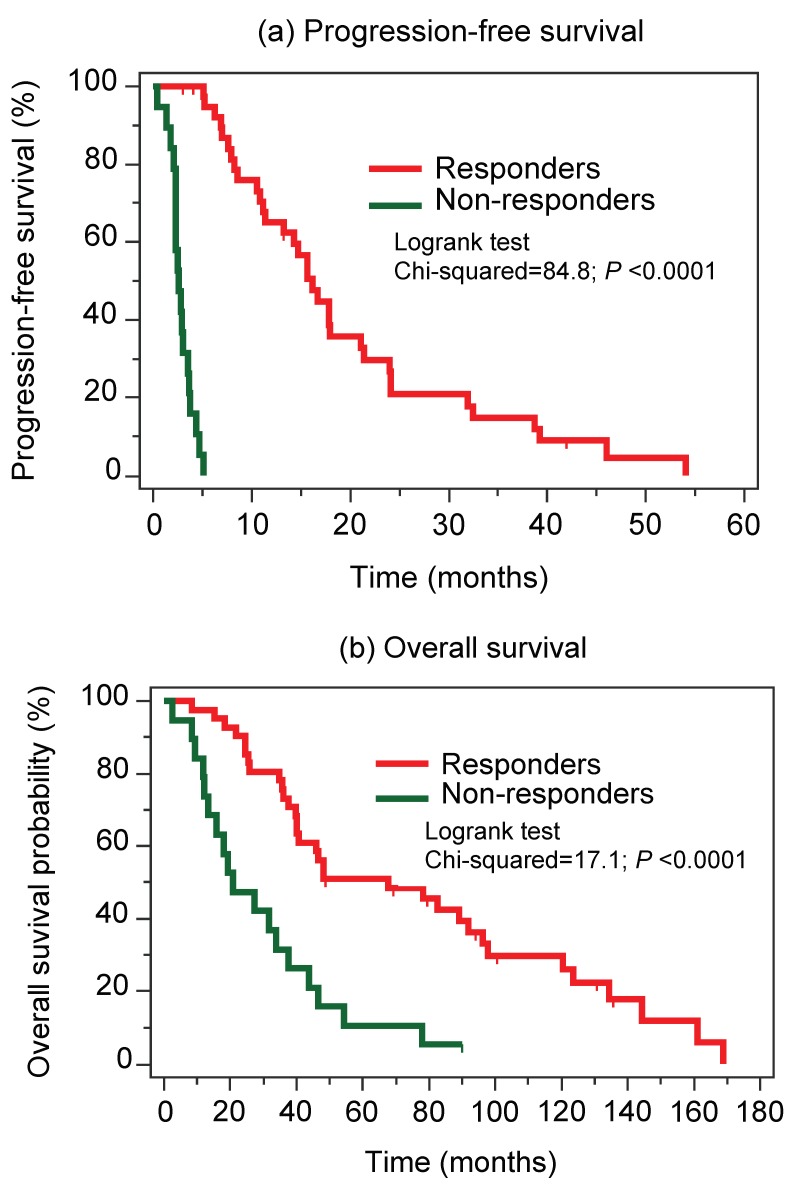
Kaplan-Meier curves in mRCC patients under first-line TKI therapy. (**a**) Progression-free survival. (**b**) Overall survival. Patients were classified as non-responders (*n* = 19) and responders (*n* = 41) as shown in Table 1 and explained in the text. The log-rank test was used to confirm significant differences between the survival probabilities. Abbreviations: mRCC, metastatic renal cell carcinoma; TKI, tyrosine kinase inhibitors.

To avoid both type I and type II errors, the conventional thresholds of α = 5% (significance level) and β = 20% (power of 80%) were selected for sample size calculation on the basis of ROC analysis. An area under the ROC curve (AUC) of 0.75 was defined as the required discrimination criterion considering a 2 to 1 ratio of responder to non-responder in the study cohort. Under this condition, a discriminative capacity could be expected by studying 30 responders and 15 non-responders in each group. Thus, primary tumor samples from 41 patients with a partial response or stable disease criteria [4,5], defined as responders and from 19 patients who showed progressive disease after the initiation of the TKI therapy within a median period of 2.6 months, defined as non-responders, were included in this retrospective study. The clinicopathological data of the patients are summarized in Table 1 and did not differ between the two groups except for their metastatic status. Kaplan-Meier analyses of progression-free survival and overall survival showed a poor clinical outcome with shorter survival rates in patients with primary resistance (Figure 2).

The purity of the isolated RNA samples did not differ between the study groups and fulfilled the purity criterion for down-stream measurements summarized in Supplementary Information S2.

### 3.2. Array-based Identification of Deregulated miRNAs

To identify putative miRNAs as predictors of primary resistance to sunitinib, we performed a miRNA TaqMan Array Card screening analysis on pools of total RNA isolated from 10 randomly selected patients from the two groups (Figure 1; Supplementary Information S1). For the detection of differentially expressed miRs, delta Cq values of the corresponding miRNAs were calculated. From 754 miRNAs on the cards, after elimination of undetermined and very poorly expressed miRNAs (Cq > 34 in both groups), 309 miRNAs remained for further calculations (Supplementary Table S1). Using a Cq difference of ≥1.5, 11 increased and 35 decreased miRNAs between non-responders and responders were identified. We selected 11 miRs (miR-9-5p, miR-20b-5p, miR-203a-3p, miR-204-5p, miR-223-3p, miR-342-3p, miR-483-5p, miR-489-3p, miR-500a-5p, miR-885-5p and miR-1269a; miRBase 22 release, http://mirbase.org) with good PCR curves on the array cards for further validation.

### 3.3. Droplet Digital RT-qPCR Validation of Selected miRNAs

To assess in a first validation step whether the assay-based selected miRNAs could also be reliably measured using individual RT-qPCR assays, we analyzed the RNA pools used in the TaqMan Array Card screening analysis on the LightCycler. MiR-342-3p and miR-1269a of the 11 abovementioned miRNAs were detected at Cq values > 34 or showing a Cq difference < 1.0 between the pools of the two groups. Because we consider these analytical conditions as not suitable for a fit-for-purpose assay, we disregarded them in the further validation process. The Cq values of the remaining nine miRNAs obtained on the TaqMan arrays and measured on the LightCycler were strongly correlated (Supplementary Figure S2). These data confirmed the feasibility of measuring these miRNAs using TaqMan assays on either the real-time LightCycler or the droplet QX200 digital instrument.

**Figure 3:**
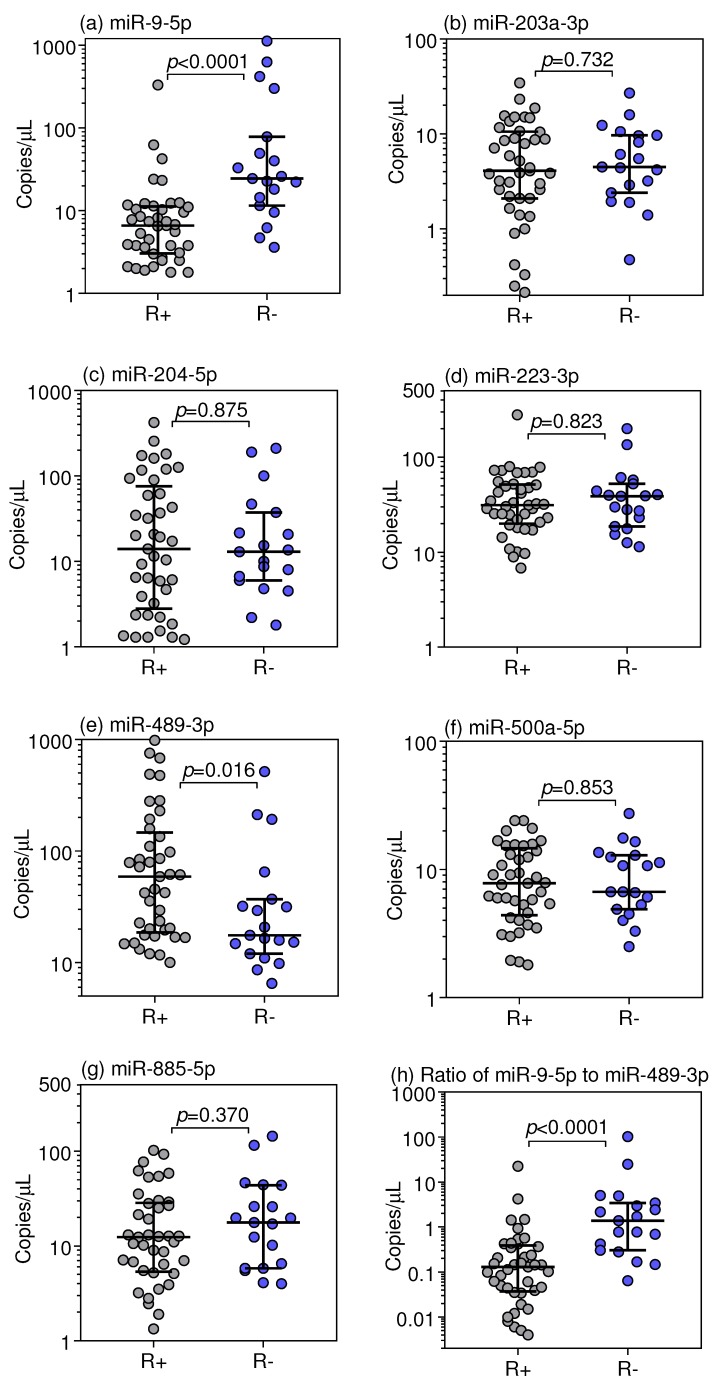
miRNA expression data in nephrectomized tumor samples of mRCC patients with different responses to first-line TKI therapy. Using droplet digital RT-PCR, the selected seven miRNAs after pretesting validation by RT-qPCR (see Figure 1 and text in Section 3.3) were measured as shown in the subfigures (**a**–**g**) in primary tumor samples of metastatic RCC patients who were responders (R+, *n* = 41) or non-responders (R−, *n* = 19) to TKI treatment. Furthermore, the ratio of the two significant miRNAs, miR-9-5p to miR-489-3p, was shown in subfigure (**h**). Medians with interquartile ranges are given, and significant differences between the study groups were calculated by Mann-Whitney *U* test. Abbreviations: mRCC, metastatic renal cell carcinoma; TKIs, tyrosine kinase inhibitors.

To take advantage of the high analytical performance of the droplet digital PCR procedure, we continued the measurements utilizing this technique. However, the assays for miR-20b-5p and miR-483-5p did not form proper droplet cluster pictures making reasonable measurements impossible. Thus, we continued the droplet digital PCR measurements with the other seven miRNAs (miR-9-5p, miR-203a-3p, miR-204-5p, miR-223-3p, miR-489-3p, miR-500a-5p, and miR-885-5p). The expression data from the patients classified as responder and non-responder patients (Table 1) are shown in Figure 3.

The level of miR-9-5p was significantly increased, whereas the level of miR-489-3p was decreased, in non-responder patients compared to that in responder patients. This differential expression effect was accentuated when the ratio of miR-9-5p to miR-489-3p was established. However, it was additionally remarkable that both miRNAs were not differentially expressed between non-malignant adjacent and malignant tissue samples (Supplementary Figure S4). This feature distinctly contrasted with the other examined miRNAs without the differential expression in the responder-dependent groups since their expression was either increased (miR-885-5p and miR-223-3p) or decreased (miR-203a-3p, miR-204-5p and miR-500a-5p) in tumor samples in comparison to non-malignant adjacent tissue samples (Supplementary Figure S4).

The clinicopathological variables pT stage, Fuhrman grade and metastatic status did not correlate with the expression of the seven miRNAs (P values between 0.162 and 0.952) except for the Fuhrman grade with miR-9-5p (r_s_ = 0.324, *p* = 0.013) and miR-223-3p (r_s_ = −0.311, *p* = 0.017) (Supplementary Table S9). Significantly different correlation coefficients of the miRNA expression in dependence on the clinicopathological variables were not observed (*p* < 0.05) between the responder and non-responder patients.

### 3.4. miRNAs as Predictors of Primary Resistance to the TKI Treatment and Clinical Outcome

**Figure 4:**
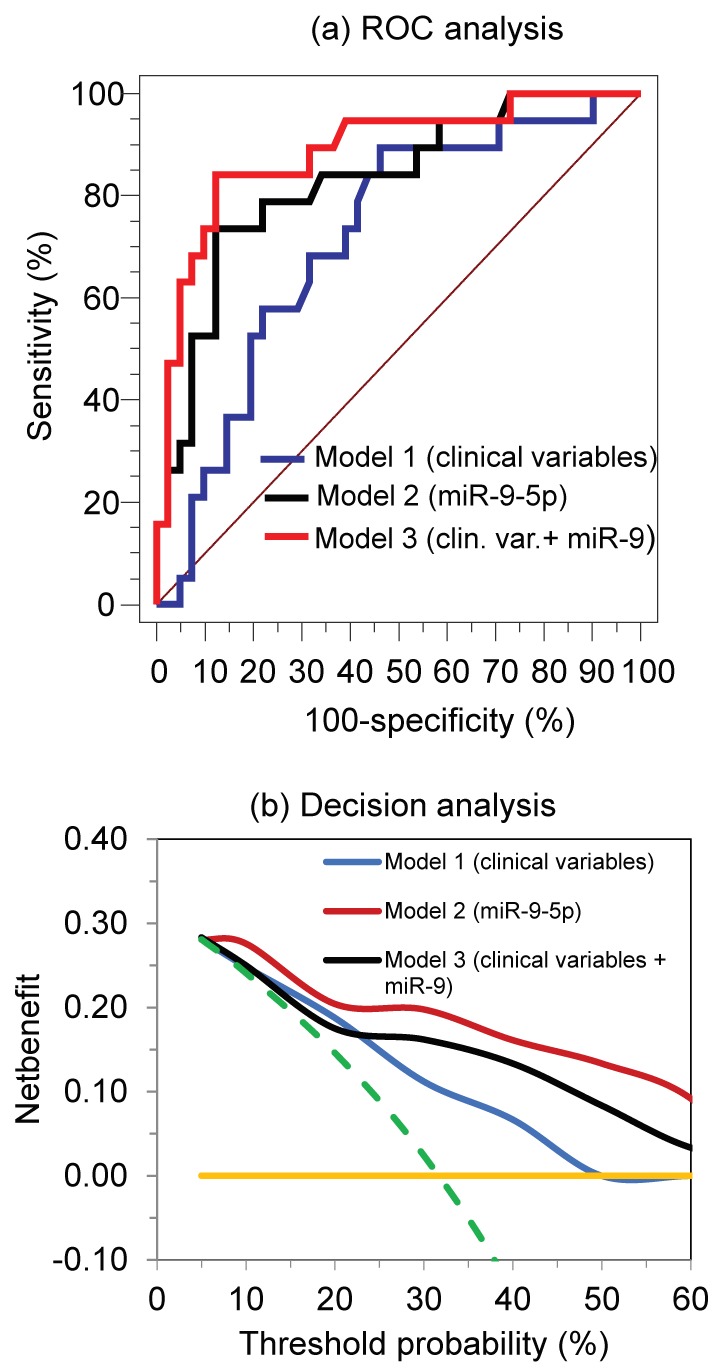
ROC curve analysis (**a**) and decision curve analysis (**b**) showing the benefit of miR-9-5p for predicting primary resistance to TKIs in mRCC patients in comparison to that for clinicopathological variables. Binary logistic regression models were calculated. Model 1 included the clinicopathological variables age, sex, tumor stage, Fuhrman grade, metastatic and resection status as indicated in Table 1; Model 2 considered only continuous miR-9-5p values; and Model 3 was a combination of the clinicopathological variables and miR-9-5p values (Model 1 + Model 2). Output data of binary logistic regression models were used for ROC and decision curve analyses as previously described [41,42]. The areas under the ROC curves (and the 95% confidence intervals) were 0.72 (0.59–0.83) for Model 1, 0.83 (0.71–0.91) for Model 2, and 0.89 (0.78–0.91) for Model 3. Abbreviations: ROC, receiver-operating characteristic; mRCC, metastatic renal cell carcinoma; TKIs, tyrosine kinase inhibitors.

ROC analysis was used to characterize the discriminative potential of the single and combined miRNAs as well as their combination with clinicopathological variables to response/non-response to TKI treatment (Table 2, Figure 4). As expected from the expression data shown in Figure 2, the single miRNAs miR-9-5p and miR-489-3p showed statistically significant AUCs. Including all miRNAs in a model built by binary logistic regression analysis or the combination of miR-9-5p and miR-489-3p in different way, a comparable discrimination between the two groups was achieved in comparison with miR-9-5p (Table 2). For practical reasons, we continued our model calculations with miR-9-5p.

Figure 4 shows the ROC curves and the decision analyses of the combined clinicopathological variables in Model 1, with miR-9-5p as the best single discriminative miRNA in Model 2 and the combination of the clinicopathological variables with miR-9-5p in Model 3. MiR-9-5p revealed a higher AUC value than that of clinicopathological variables of Model 1 and improved the discrimination ability in combination with the clinical variables (Figure 4a). In the respective decision curve analysis (Figure 4b), this net benefit of miR-9-5p was also displayed compared to that of the use of clinicopathological variables as predictors of primary resistance to the TKI treatment.

**Figure 5:**
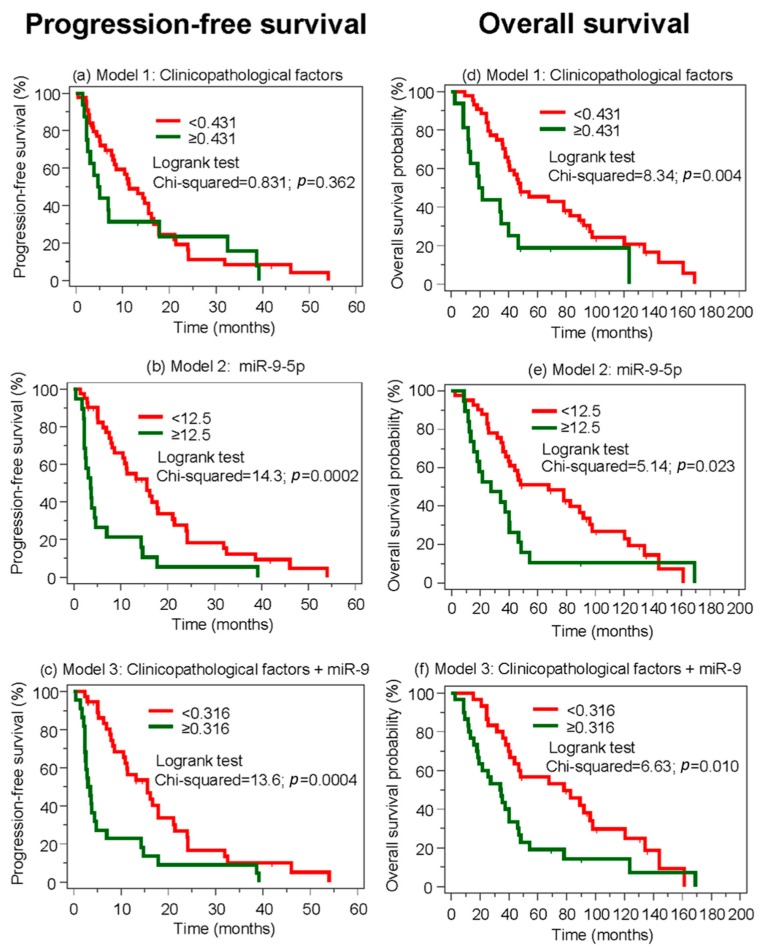
Kaplan-Meier curves of progression-free survival and overall survival based on the prediction models of primary resistance to the treatment with TKIs. The variables of the prediction models were used in the binary logistic regression analyses. Model 1 included the clinicopathological variables age, sex, tumor stage, Fuhrman grade, metastatic and resection status as indicated in Table 1; Model 2 considered only continuous miR-9-5p values; and Model 3 was a combination of the clinicopathological and miR-9-5p values. The propensity scores of logistic regression analyses as outputs of the predicted probabilities were used to calculate the best discriminatory cutoffs using the Youden index in ROC analysis. Based on these cutoffs indicated in the respective figures, the output data were dichotomized and the corresponding Kaplan-Meier curves for progression-free survival (left column, subfigures (**a**–**c**)) and overall survival (right column, subfigures (**d**–**f**)) were generated. TKI, tyrosine kinase inhibitors.

It was furthermore of interest whether miR-9-5p in these three prediction models for the primary resistance could also be used as a predictive marker for progression-free and overall survivals. Comparison of the Kaplan-Meier curves of progression-free survival according to the conventional clinicopathological variables and miR-9-5p supports this view (Figure 5a–c). These analyses demonstrate that the combination of conventional clinicopathological parameter data with the most significant miR-9-5p could improve the reliability of forecasting primary resistance to TKIs. In the multivariate Cox regression model of progression-free survival with all clinicopathological variables, miR-9-5p showed a hazard ratio of 3.23 (95% CI 1.67 to 6.28; *p* = 0.0005) and remained as the only significant factor after a backward elimination process. For the prediction of overall survival, the inclusion of miR-9-5p in the model with the clinicopathological variables showed merely a rather adjunctive role (Figure 5d–f).

Using the miRTarBase, miRWalk2.0 and DIANA-TarBase v.7.0 databases with regard to experimentally validated miRNA-gene interactions [43,44,45], 310 target genes for miR-9-5p (Supplementary Table S10) and one target for miR-489 (PTPN11, protein tyrosine phosphatase, non-receptor type 11) have been documented so far.

## 4. Discussion

This study identified the expression of increased miR-9-5p and decreased miR-489-3p in nephrectomy specimens of RCC patients as predictive biomarkers of primary resistance to the first-line treatment with tyrosine kinase inhibitors. As the combination of both miRNAs did not improve the predictive ability of miR-9-5p, we focused on data evaluation of miR-9-5p for practical purposes.

The identification of novel biomarkers that could be informative for therapeutic efficacy, safety and tolerability of targeted drugs is of general interest and, at the same time, a great challenge [19]. Despite the development and wide use of targeted therapies for mRCC, no molecular marker is currently applied in practice for these predictive purposes in the management of mRCC patients [17]. This lack of markers applies not only to tissue markers but also to circulating markers from blood or plasma/serum [46,47], as currently summarized in two overviews [9,17].

Our results confirm other study data regarding miRNAs as helpful tools for this current issue in the application of TKIs in the first-line treatment of mRCC patients [21,22,23,24,25,26,27,48]. However, the different results between those studies and our data should be mentioned. Three studies exclusively examined ccRCC samples [21,22,23], and another as well as our study included approximately 10% papillary RCC [24]. Similar to our approach, five working groups used a two-stage design to identify potential predictive miRNAs in nephrectomy specimens. Such an approach consisted of an initial array- or sequencing-based screening step to discover deregulated miRNAs associated with a therapeutic response to a TKI in either fresh-frozen RCC or FFPE RCC samples followed by a second RT-qPCR validation step for selected miRNAs [21,22,23,24,25]. Moreover, Gamez-Pozo et al. [48] examined miRNAs in peripheral blood samples from mRCC patients as predictors of resistance to sunitinib. All of these studies revealed significant relationships between miRNAs and progression-free survival as an indicator of the response to sunitinib, similar our study cohort treated with sunitinib and the two other guideline-approved TKIs, pazopanib and sorafenib. However, different miRNAs were recommended by all of the studies as best predictors of resistance to sunitinib. Kovacova et al. [27] confirmed this less than satisfactory situation. The authors aimed to validate miRNAs identified in previous studies as predictive markers of response to sunitinib therapy, but only two out of 10 examined miRNAs were shown to be discriminative between responders and non-responders in their study cohort.

Preanalytical and analytical variations might be one reason for the different results observed between the studies. However, the selected time interval after the initiation of TKI-based therapy for evaluating the response and, in consequence, stratification of the patients into corresponding cohorts, likely had much more influence on the expression differences [28]. Our approach was clearly focused on primary resistance [8,9] to identify miRNA expression characteristics in nephrectomy specimens, which could be indicative in predicting therapeutic efficacy for the respective patient. This approach could be a basis for a companion diagnostic test performed before therapy is initiated to prove whether the patient could benefit from this treatment or not [49]. Therefore, we defined patients with progressive disease in the first restaging visit after the start of targeted therapy (median: 2.6 months) and compared their expression data with patients who suffered progressive disease much later (median: 16.2 months). This approach corresponds to the stratification concept applied in the studies by Berkers et al. [21] and Prior et al. [22], who also used similar extreme endpoints (2.7 vs. 24.2 and 2–3 vs. 31–32 months). Other studies stratified the patients being treated with sunitinib into cohorts according to an endpoint of progressive-free survival for 9 or 12 months under sunitinib [23,24]. By using those time criteria, it cannot be excluded that the non-responder group defined in such a way could also include patients with a secondary resistance, which is often observed 6 months after initiation of sunitinib treatment [7].

Based on the 46 differentially expressed miRNAs in our array-based screening process, we decided to use droplet digital RT-PCR as favorable analytical method for further quantification of the seven selected miRNAs. The droplet digital RT-PCR technique enables quantification of the absolute number of transcripts and therefore circumvents a potentially erroneous normalization procedure [25]. Although the model with all seven miRNAs achieved a perfect discrimination between the two cohorts (Table 2), we did not use this model to avoid the risk of overfitting bias. Our study intention was to improve the basic predictive ability of clinicopathological data by combining them with the best quantitative biomarkers according to the principle of “do not use more laboratory tests than absolutely necessary” [50]. The use of a single marker, miR-9-5p, the ratio of miR-9-5p to miR-489-3p and their combination with clinicopathological variables in binary logistic regression models resulted in a similar high discrimination capacity and confirmed our strategy (Table 2, Figure 4a). Thus, a focus on the best discriminative miRNA miR-9-5p was feasible and justified. This result is also clearly shown in the decision curve analysis complementing the ROC curves (Figure 4a,b). Decision-curve analysis is a helpful decision-making tool when different markers should be compared with regard to their clinical value [41]. The increased net benefit of miR-9-5p alone and in combination with the clinicopathological variables becomes obvious over a wide range of threshold probabilities in comparison to the model with the clinicopathological variables alone (Figure 4b).

The missing correlations of miRNAs with the clinicopathological risk factors pT stage, Fuhrman grade and metastatic status, especially those of the two differentially expressed miR-9-5p and miR-489-3p in responders and non-responders, are remarkable. This uncorrelated differential expression of miRNAs in relation to these known disease variables is a striking hallmark of potential orthogonal biomarkers [51]. This particularity of orthogonal markers must be regarded as a precondition that these markers are independent factors in a prognostic model. These independent variables in a multivariable model adjusted by clinicopathological factors are frequently associated with the subsequent discovery of novel downstream targets and pathways [51]. Our study proved that miR-9-5p remained such an independent variable in multivariable Cox regression models (Figure 5). This kind of marker could improve recently used prognostic models to guide the treatment choices of patients [12,13,14].

The identified differentially expressed miRNAs in TKI-non-responsive patients are not only useful as predictive biomarkers. Since miRNAs have fundamental regulatory functions in various signal cascades and metabolic pathways, it can be assumed that they can be helpful for a deeper understanding of the underlying mechanisms involved in primary resistance to TKIs. MiR-9-5p is the major transcript from the three genes *MIR9-1*, *MIR9-2* and *MIR9-3* that are located on chromosomes 1, 5 and 15. Increased expression of miR-9 was found in liver carcinoma [52,53], breast cancer [54] and esophageal carcinoma [55], while decreased expression was described in colorectal carcinoma [56], gastric and ovarian cancer [57,58]. These different expression characteristics imply that the miRNA has cancer-specific functional roles. A few reports exist about the expression of miR-9-5p in ccRCCs [59,60,61]. Specific expression data of miR-9 in RCC tissue and cancer cell lines have been reported in relation to methylation status with divergent results [59,61]. Thus, the significance of this miRNA with regard to the sunitinib resistance as observed in our expression data can only be deduced from the functional data explored in other tumor entities. Experimentally validated interactions of miR-9 with 310 genes (Supplementary Table S10) have been documented so far in the miRTarBase, miRWalk2.0 and DIANA-TarBase databases [43,44,45]. Several of these genes are involved in cancer-related pathways such as VEGF, angiogenesis, apoptosis and E-cadherin, as shown in the KEGG pathway database (http://www.kegg.jp/). For example, in breast cancer and esophageal carcinoma, miR-9 has been found to promote epithelial-mesenchymal transition as an important mechanism in tumor progression [54,55,62]. In addition, it is of interest regarding the new therapy concept of immune checkpoint blockade using antibodies against PD-1 that miR-9 is involved, together with its target gene *PRDM1* (PR domain containing 1, with ZNF domain) and cytokine IL-2, in T-cell dysfunction [63,64]. For miR-489, only one validated interaction with PTPN11 was documented in the three databases. Increased expression of PTPN11 is currently postulated to be a key element in intrinsic and acquired resistance to other targeted cancer drugs [65]. However, the relationships to TKI therapy in mRCC patients have not yet been examined.

Limitations of this study must be mentioned. The genetic heterogeneity of RCCs is a general inherent limitation of this tissue-based study [66]. This heterogeneity between the primary RCC and its metastatic lesions is also a further reason for the expression differences observed between different studies as well as between screening and validation cohorts within the same study [22,24]. To overcome this difficulty of heterogeneity, the examination of multiple samples or several mixed samples from the same tumor has been suggested [23,67]. The more specific limitations of our study are its retrospective design and the lack of external validation of the data. Under the aspect of the selected strict rules to assemble two different patient cohorts under TKI treatment, the retrospective design for this pilot study should be considered acceptable. In this regard, all analyses were therefore performed in a blinded manner before the data were statistically evaluated. To exclude type I and II errors as far as possible, we followed a sample size calculation with a high effect size as explained. Moreover, the additional digital PCR analyses and the approach of internal validation of data by bootstrapping in addition to the decision curve analyses as shown in our previous studies of biomarkers [33,50] support the validity of our study. A real limitation of our study is the lack of functional experiments to show the potential effects of miR-9-5p and miR-489-3p in association with primary resistance. However, investigation of the functional role of these miRNAs was beyond the scope of this study as we pointed out the aims of this study in the Introduction. In addition, the functional role of a biomarker may be helpful to explain its biological rationale, but is not pivotal for its application in clinical practice to prove its clinical benefit in comparison to the currently used decision-making procedures [68].

## 5. Conclusions

In conclusion, miRNA expression patterns in nephrectomy specimens might be helpful in predicting primary resistance to first-line TKI treatment in mRCC patients. In this study, miR-9-5p alone and in combination with conventional clinicopathological factors could improve the objectivity of the decisions of clinicians and patients in individualized patient counseling in determining whether the patient would benefit from the drug or would fail to respond to the drug. In addition, the inclusion of predictive biomarkers into prognostic models could improve the accuracy and robustness of those models. Considering the findings and critical points discussed in this article, all of these results make further prospective studies worthwhile. This should also include experimental work regarding the possible functional role of miRNAs in primary resistance to first-line TKI treatment in mRCC patients.

## Figures and Tables

**Table 1:** Clinicopathological characteristics of the metastatic renal cell carcinoma cohort after radical nephrectomy classified as responder and non-responder patients to a first-line tyrosine kinase inhibitor therapy.

Patient Characteristics	TKI Therapy Groups		*p* Value *
	Responders	Non-Responders	
Number	41	19	
Sex			0.081
female	13	2	
male	28	17	
Age, median years (95% CI)	62.0 (59.0–67.2)	59.0 (53.6–67.0)	0.427
Tumor characteristics			0.678
clear cell carcinoma	38	17	
papillary carcinoma	3	2	
Tumor stage			0.226
pT1	8	4	
pT2	5	-	
pT3	25	15	
pT4	3	-	
Regional/distant metastasis ^†^			0.033
pN0/M0	23	5	
pN1/M1	18	14	
Residual tumor			0.528
R0/Rx	32	16	
R1/2	9	3	
Fuhrman grade			0.229
FG1/2	25	9	
FG3/4	14	10	
FGx ^‡^	2	-	
First-line TKI therapy			0.313
Sunitinib	35	16	
Pazopanib	3	3	
Sorafenib	3	-	
Response to targeted therapy			
Partial response	11	-	
Stable disease	30	-	
Progressive disease	-	19	
Median months survival (95% CI) ^§^			
Progression-free	16.2 (11.4–21.0)	2.6 (2.3–3.5)	<0.0001
Overall	67.6 (40.1–96.2)	20.9 (13.3–37.4)	<0.0001

Abbreviations: CI, confidence interval; FG, histopathological grading according to Fuhrman; M, distant metastasis; pN, pathological nodal status; pT, pathological tumor classification; R, surgical margin classification; TKI, tyrosine kinase inhibitors. * *p* values from Fisher’s exact test, Chi-square test or Mann-Whitney *U* test indicate the association of the responder status with the clinicopathologic characteristics in the patients. ^†^ Imaging techniques was used to assess the presence/non-presence of metastases (M1/M0) before surgery. ^‡^ Unclassified patients. ^§^ Data were taken from the Kaplan-Meier analyses. The median survival corresponds to the time at which the survival probability is 50%.

**Table 2:** Receiver-operating characteristic curve analyses of miRNAs in nephrectomized tumor tissue samples to discriminate between patients as responders and non-responders to the treatment with tyrosine kinase inhibitors.

miRNA	AUC (95% CI)	*p* Value Different to AUC = 0.5	Differentiating Ability at the Youden Index *		Overall Correct Classification (%)
			Sensitivity (%)	Specificity (%)	
*Single miRNAs*					
miR-9-5p	0.83 (0.71–0.91)	<0.0001	74	88	73
miR-203a-3p	0.53 (0.40–0.66)	0.718	63	51	68
miR-204-5p	0.51 (0.38–0.65)	0.861	89	29	68
miR-223-3p	0.52 (0.03–0.65)	0.871	53	61	68
miR-489-3p	0.69 (0.58–0.80)	0.014	79	59	68
miR-500a-5p	0.52 (0.38–0.65)	0.846	16	73	68
miR-885-5p	0.57 (0.44–0.70)	0.353	58	63	72
*Combined miRNAs*					
All miRNAs ^†^	0.79 (0.66–0.88)	<0.0001	95	59	77
miR-9/miR-489	0.83 (0.73–0.93)	<0.0001	69	87	73
miR-9 + miR-489 ^†^	0.82 (0.71–1.00)	<0.0001	97	21	72

Abbreviations: AUC, area under the receiver-operating characteristics curve; CI, confidence interval. * The cutoff at the maximum value of the Youden index corresponding to the overall diagnostic effectiveness with the equal weight to false positive and false negative values was used for this assessment. ^†^ Calculated by full binary logistic regression.

## Supplementary Materials

The following are available online at http://www.mdpi.com/2072-6694/10/9/321/s1, Information S1: TaqMan® Array Human MicroRNA Cards for discovery and miRNA selection for validation, Information S2: Methodologies of RT-qPCR and digital PCR, Figure S1. Characteristics of PCR standard curves, Figure S2. Correlation between Array data and RT-qPCR data, Figure S3. Fluorescence scatter plots of the dd PCR assays, Figure S4. Expression of miRNAs in tumor samples of RCC patients compared to normal adjacent renal parenchyma. Table S1. TaqMan MicroRNA Array data ranked according to Cq differences, Table S2. MIQE checklist according to Bustin et al., Table S3. Digital MIQE checklist according to Huggett et al, Table S4. TaqMan miRNA assays for RT-qPCR and dd PCR, Table S5. Reproducibility of miRNA measurements, Table S6. Measurement conditions of dd PCR analyses, Table S7. Parameter λ given as median copies per partition, Table S8. Repeatability and reproducibility data, Table S9. Spearman rank correlations between pathological variables and miRNAs, Table S10. List of validated target genes of miR-9-5p.
