# Supplementary material for: miR-9-5p in Nephrectomy Specimens is a Potential Predictor of Primary Resistance to First-Line Treatment with Tyrosine Kinase Inhibitors in Patients with Metastatic Renal Cell Carcinoma
Source: Cancers (Basel). 2018 Sep 10;10(9):321. doi: 10.3390/cancers10090321 (PMC6162741; doi:10.3390/cancers10090321)
Supplement: Supplementary file 1 [file cancers-10-00321-s001.pdf]

Supplementary Materials: miR-9-5p in Nephrectomy Specimens is a Potential Predictor of Primary Resistance to First-Line Treatment with Tyrosine Kinase Inhibitors in Patients with Metastatic Renal Cell Carcinoma

Bernhard Ralla Jonas Busch, Anne Flörcken, Jörg Westermann, Zhongwei Zhao, Ergin Kilic [3], Sabine Weickmann, Monika Jung, Annika Fendler and Klaus Jung

Information S1: TaqMan® Array Human MicroRNA Cards for discovery and miRNA selection for validation S2
Table S1. TaqMan MicroRNA Array data ranked according to Cq differences S2
Information S2: Methodologies of RT-qPCR and digital PCR S9
General comments regarding the PCR guidelines and RNA quality data S9
Table S2. MIQE checklist according to Bustin et al. S9
Table S3. Digital MIQE checklist according to Huggett et al. S13
Pre-testing step: RT-qPCR analyses with the LightCycler 480 .... S16
Table S4. TaqMan miRNA assays for RT-qPCR and dd PCR S16
cDNA synthesis S17
Quantitative real-time PCR S17
Performance data of RT-qPCR analyses S17
Figure S1. Characteristics of PCR standard curves S18
Table S5. Reproducibility of miRNA measurements S19
Figure S2. Correlation between Array data and RT-qPCR data S19
Validation step: Droplet digital PCR with the QX200 digital PCR instrument S20
Methodical details of the droplet digital PCR S20
Table S6: Measurement conditions of dd PCR analyses S20
Figure S3. Fluorescence scatter plots of the dd PCR assays S22
Table S7. Parameter λ given as median copies per partition S24
Performance data of the droplet digital PCR analyses S25
Table S8. Repeatability and reproducibility data S25
Expression and correlation data S26
Figure S4. Expression of miRNAs in tumor samples of RCC patients compared to normal adjacent renal parenchyma S26
Table S9. Spearman rank correlations between pathological variables and miRNAs S27
Table S10. List of validated target genes of miR-9-5p S27
References in the supplementary materials S33

Information S1: TaqMan® Array Human MicroRNA Cards for discovery and miRNA selection for validation.

As described in Materials and methods, TaqMan® Array Human MicroRNA A+B Cards Set v3.0 (Thermo Fisher Scientific, Waltham, MA, USA; Cat. No: 4444913) were used in the first step for the identification of differentially expressed miRNAs in primary RCC tissue samples depending on the response status to TKIs. In total, 754 human miRNAs and 4 snRNAs as multi-controls can be determined on these two microfluidic cards (A and B). For that purpose, two RNA sample pools consisting of equal RNA aliquots from ten sensitive (responders) and ten resistant (non-responders) patients to the sunitinib treatment were prepared. A multiplexed cDNA synthesis was performed. One µg RNA per three µL RNA pool was reverse-transcribed with components of the TaqMan MicroRNA Reverse Transcription Kit (Thermo Fisher; Cat. No. 4366596) and the Megaplex RT Primers, Human Pools Set v3.0 (Cat. No. 4444745) with pool A v2.1 and pool B v3.0. Tubes with a final megaplexed RT reaction volume of 7.5 µL were incubated in a Block Thermal cycler (Biometra, Göttingen, Germany) according to the Megaplex Pools Protocol without pre-amplification step (Thermo Fisher; PN 4399721). Then, six µL of the megaplex-pool-specific cDNAs were mixed with TaqMan® Universal PCR Master Mix, No AmpErase® UNG, 2x (Cat. No. 4364343) and nuclease-free water (5Prime, Hamburg, Germany, Cat. No. 2500000) to a final volume of 900 µL. Each of the 8 ports of the TaqMan MicroRNA array card was filled with 100 µL PCR reaction mix. After array centrifugation and sealing, the arrays were measured on the real-time PCR system ViiA7 (Thermo Fisher Scientific) under default thermal-cycling conditions for the 384 well TaqMan microRNA array cards. Cq values were generated by the SDS software v2.3 and were exported for further calculations. For the detection of differentially expressed miR, delta Cqs of the corresponding miRNAs were calculated. Undetermined and very low expressed miRNAs (Cq >34.0 in both groups), were eliminated so that 309 miRNAs remained for further calculations. These miRNAs were ranked according to the Cq differences between both groups summarized in Table S1. Using a Cq difference of ≥1.5 corresponding a fold change of 2.82, 11 up- and 35 down-regulated miRNAs between non-responders and responders were identified. We selected 11 miRs (miR-9-5p, miR-20b-5p, miR-203a-3p, miR-204-5p, miR-223-3p, miR-342-3p, miR-483-5p, miR-489-3p, miR-500a-5p, miR-885-5p, and miR-1269a; miRBase 22 release, http://mirbase.org) with good PCR curves on the array cards for further validation.

Supplementary Table S1. TaqMan MicroRNA Array data ranked according to the Cq-value differences between sunitinib non-responders and responders. The selected miRNAs according to the mentioned criteria used in the first validation step running on the LightCycler are marked in the last column.

**miR-Name AB-Assay ID**	**Cq-Value Responder (R)**	**Cq-Value Non-Responder**	**Delta Cq R-(non-R)**	**miR-Regulation (Non-R to R) One-Sided with Cq<33**	**Assays Run on LC480**
hsa-miR-9-000583	31.26	26.79	**4.5**	up	**X**
hsa-miR-583-001623	36.91	32.79	**4.1**	up	**-**
hsa-miR-9#-002231	34.20	30.70	**3.5**	up	**-**
hsa-miR-483-5p-002338	32.60	30.49	**2.1**	up	**X**
hsa-miR-125a-3p-002199	35.10	33.17	**1.9**	up	**-**
hsa-miR-338-3p-002252	33.97	32.12	**1.9**	up	**-**
hsa-miR-483-3p-002339	31.17	29.32	**1.9**	up	**-**
hsa-miR-584-001624	33.55	31.70	**1.8**	up	**-**
hsa-miR-885-5p-002296	31.82	30.11	**1.7**	up	**X**
hsa-miR-1247-002893	30.43	28.83	**1.6**	up	**-**
hsa-miR-223-002295	24.66	23.12	**1.5**	up	**X**
hsa-miR-1300-002902	32.56	31.16	1.4	up	
hsa-miR-643-001594	33.44	32.24	1.2	up	
hsa-miR-1225-3P-002766	31.73	30.53	1.2	up	

miR-Name AB-Assay ID	Cq-Value Responder (R)	Cq-Value Non-Responder	Delta Cq R-(non-R)	miR-Regulation (Non-R to R) One-Sided with Cq<33	Assays Run on LC480
hsa-miR-941-002183	33.43	32.26	1.2	up	
hsa-miR-431-001979	34.50	33.34	1.2	up	
hsa-miR-539-001286	34.52	33.38	1.1	up	
hsa-miR-20a#-002437	34.11	32.98	1.1	up	
hsa-miR-1248-002870	33.79	32.67	1.1	up	
hsa-miR-212-000515	33.99	32.89	1.1	up	
hsa-miR-143#-002146	31.32	30.24	1.1	up	
hsa-miR-638-001582	32.92	31.87	1.1	up	
hsa-miR-338-5P-002658	34.30	33.30	1.0	up	
hsa-miR-769-5p-001998	31.97	30.97	1.0	up	
hsa-miR-181a-2#-002317	32.52	31.53	1.0	up	
hsa-miR-572-001614	34.40	33.41	1.0	up	
hsa-miR-625-002431	34.12	33.21	0.9	up	
hsa-miR-1228#-002763	30.17	29.27	0.9	up	
hsa-miR-301b-002392	34.75	33.85	0.9	up	
hsa-miR-193b#-002366	34.38	33.50	0.9	up	
hsa-miR-199a-000498	34.18	33.40	0.8	up	
hsa-let-7e-002406	27.71	26.97	0.7	up	
hsa-miR-566-001533	30.80	30.06	0.7	up	
hsa-miR-142-3p-000464	26.18	25.49	0.7	up	
hsa-miR-424-000604	32.67	31.98	0.7	up	
hsa-miR-511-001111	34.56	33.88	0.7	up	
hsa-miR-200c-002300	30.22	29.55	0.7	up	
hsa-miR-190b-002263	34.09	33.43	0.7	up	
hsa-miR-636-002088	33.55	32.89	0.7	up	
hsa-miR-376a-000565	33.08	32.43	0.7	up	
hsa-miR-324-3p-002161	30.07	29.43	0.6	up	
hsa-miR-378-000567	34.02	33.43	0.6	up	
hsa-miR-376c-002122	30.22	29.65	0.6	up	
hsa-miR-144#-002148	32.52	31.98	0.5	up	
hsa-miR-29a#-002447	33.94	33.40	0.5	up	
hsa-miR-519e#-001166	33.47	32.95	0.5	up	
hsa-miR-199a-3p-002304	27.72	27.20	0.5	up	
hsa-miR-577-002675	34.10	33.59	0.5	up	
hsa-miR-98-000577	33.23	32.73	0.5	up	
hsa-miR-1275-002840	28.81	28.31	0.5	up	
hsa-miR-1255B-002801	33.33	32.83	0.5	up	
hsa-miR-328-000543	32.31	31.86	0.4	up	
hsa-miR-142-5p-002248	31.93	31.49	0.4	up	
hsa-miR-659-001514	33.05	32.62	0.4	up	
hsa-miR-192#-002272	32.70	32.27	0.4	up	
hsa-miR-1291-002838	32.75	32.33	0.4	up	
hsa-miR-335#-002185	32.63	32.23	0.4	up	
hsa-miR-661-001606	29.08	28.70	0.4	up	
hsa-miR-24-2#-002441	33.91	33.55	0.4	up	
hsa-miR-425-5p-001516	29.25	28.90	0.4	up	
hsa-miR-1285-002822	31.33	31.00	0.3	up	
hsa-miR-125b-000449	26.76	26.42	0.3	up	
hsa-miR-592-001546	31.09	30.76	0.3	up	
hsa-miR-502-3p-002083	34.24	33.92	0.3	up	

miR-Name<br>AB-Assay ID	Cq-Value<br>Responder (R)	Cq-Value<br>Non-Responder	Delta Cq<br>R-(non-R)	miR-Regulation<br>(Non-R to R)<br>One-Sided with<br>Cq<33	Assays Run<br>on LC480
hsa-miR-15b#-002173	31.38	31.06	0.3	up	
hsa-miR-1270-002807	32.91	32.60	0.3	up	
hsa-miR-214-002306	28.86	28.55	0.3	up	
hsa-miR-411-001610	32.68	32.40	0.3	up	
hsa-miR-301-000528	31.35	31.09	0.3	up	
hsa-miR-155-002623	25.97	25.71	0.3	up	
hsa-miR-423-5p-002340	31.39	31.14	0.3	up	
hsa-miR-200b-002251	27.02	26.78	0.2	up	
hsa-let-7c-000379	31.65	31.42	0.2	up	
hsa-miR-150-000473	26.19	25.97	0.2	up	
hsa-miR-145#-002149	32.36	32.14	0.2	up	
hsa-miR-34a-000426	25.84	25.62	0.2	up	
hsa-miR-22#-002301	33.05	32.85	0.2	up	
hsa-miR-1227-002769	33.55	33.36	0.2	up	
hsa-miR-22-000398	25.98	25.79	0.2	up	
hsa-miR-24-000402	22.45	22.29	0.2	up	
hsa-miR-21-000397	22.47	22.32	0.2	up	
hsa-miR-370-002275	33.41	33.27	0.1	up	
hsa-let-7b-002619	26.59	26.45	0.1	up	
hsa-miR-136#-002100	34.04	33.91	0.1	up	
hsa-miR-33a#-002136	33.17	33.05	0.1	up	
hsa-miR-224-002099	30.23	30.10	0.1	up	
hsa-miR-19b-000396	23.34	23.24	0.1	up	
hsa-miR-106a-002169	24.32	24.23	0.1	up	
mean RNU44-001094	25.83	25.74	0.1	up	
hsa-let-7i#-002172	32.85	32.77	0.1	up	
hsa-miR-1253-002894	32.19	32.11	0.1	up	
hsa-miR-1274A-002883	22.41	22.33	0.1	up	
hsa-miR-346-000553	32.38	32.30	0.1	up	
hsa-miR-574-3p-002349	28.70	28.63	0.1	up	
hsa-miR-28-000411	29.29	29.25	0.0	=	
hsa-miR-628-5p-002433	32.01	31.96	0.0	=	
hsa-miR-15a-000389	28.66	28.62	0.0	=	
hsa-miR-218-000521	29.46	29.43	0.0	=	
hsa-miR-1183-002841	31.79	31.76	0.0	=	
hsa-miR-183#-002270	33.90	33.87	0.0	=	
hsa-miR-99b#-002196	32.88	32.86	0.0	=	
hsa-miR-31-002279	26.49	26.48	0.0	=	
hsa-miR-365-001020	30.64	30.62	0.0	=	
hsa-miR-151-3p-002254	26.63	26.63	0.0	=	
hsa-miR-505#-002087	32.85	32.85	0.0	=	
hsa-miR-939-002182	25.88	25.88	0.0	=	
hsa-miR-652-002352	30.33	30.33	0.0	=	
hsa-miR-19b-1#-002425	33.94	33.97	0.0	=	
hsa-miR-144-002676	31.77	31.80	0.0	=	
hsa-miR-135b-002261	28.27	28.30	0.0	=	
hsa-miR-214#-002293	32.17	32.20	0.0	=	
hsa-miR-1303-002792	33.13	33.17	0.0	=	
hsa-miR-494-002365	24.89	24.95	−0.1	down	
hsa-let-7g-002282	27.35	27.42	−0.1	down	

miR-Name<br>AB-Assay ID	Cq-Value<br>Responder (R)	Cq-Value<br>Non-Responder	Delta Cq<br>R-(non-R)	miR-Regulation (Non-R to R) One-Sided with Cq<33	Assays Run on LC480
hsa-miR-185-002271	30.18	30.25	−0.1	down	
hsa-miR-34b-002102	32.85	32.92	−0.1	down	
hsa-miR-126#-000451	26.67	26.75	−0.1	down	
hsa-miR-1233-002768	28.24	28.32	−0.1	down	
hsa-miR-766-001986	30.38	30.46	−0.1	down	
hsa-miR-335-000546	32.31	32.40	−0.1	down	
hsa-miR-191-002299	24.85	24.94	−0.1	down	
hsa-miR-486-001278	30.14	30.24	−0.1	down	
hsa-miR-424#-002309	32.13	32.25	−0.1	down	
hsa-let-7g#-002118	33.42	33.57	−0.1	down	
hsa-miR-532-3p-002355	31.17	31.33	−0.2	down	
hsa-miR-200a-000502	27.46	27.63	−0.2	down	
hsa-miR-125a-5p-002198	27.72	27.88	−0.2	down	
hsa-miR-15b-000390	30.31	30.50	−0.2	down	
hsa-miR-99b-000436	28.75	28.94	−0.2	down	
hsa-miR-193a-3p-002250	31.55	31.75	−0.2	down	
hsa-miR-95-000433	31.39	31.59	−0.2	down	
hsa-miR-16-1#-002420	33.22	33.42	−0.2	down	
hsa-miR-10a-000387	28.59	28.79	−0.2	down	
hsa-miR-148a-000470	29.10	29.31	−0.2	down	
hsa-miR-145-002278	25.43	25.65	−0.2	down	
hsa-miR-17-002308	24.24	24.46	−0.2	down	
hsa-miR-509-5p-002235	32.51	32.73	−0.2	down	
mean U6 snRNA-001973	19.86	20.11	−0.3	down	
hsa-miR-122-002245	30.44	30.71	−0.3	down	
hsa-miR-487a-001279	31.71	31.98	−0.3	down	
hsa-miR-664-002897	28.36	28.63	−0.3	down	
hsa-miR-19a-000395	26.43	26.72	−0.3	down	
hsa-miR-146b-001097	25.72	26.03	−0.3	down	
hsa-miR-186#-002105	32.39	32.71	−0.3	down	
hsa-miR-130b-000456	31.07	31.39	−0.3	down	
hsa-miR-193a-5p-002281	30.14	30.47	−0.3	down	
hsa-miR-590-5p-001984	28.91	29.24	−0.3	down	
hsa-miR-497-001043	30.93	31.26	−0.3	down	
hsa-miR-28-3p-002446	27.45	27.79	−0.3	down	
hsa-let-7a-000377	30.01	30.35	−0.3	down	
hsa-miR-34a#-002316	31.97	32.31	−0.3	down	
hsa-miR-126-002228	21.54	21.89	−0.3	down	
hsa-miR-143-002249	25.94	26.30	−0.4	down	
hsa-miR-596-001550	27.16	27.52	−0.4	down	
hsa-miR-106b-000442	27.50	27.85	−0.4	down	
hsa-miR-331-000545	26.30	26.66	−0.4	down	
hsa-miR-140-3p-002234	28.79	29.16	−0.4	down	
hsa-miR-18b-002217	32.19	32.56	−0.4	down	
hsa-miR-1201-002781	32.62	32.99	−0.4	down	
hsa-miR-133a-002246	29.98	30.36	−0.4	down	
hsa-miR-152-000475	28.42	28.80	−0.4	down	
hsa-miR-103-000439	28.88	29.27	−0.4	down	
hsa-miR-454-002323	27.33	27.72	−0.4	down	
hsa-miR-141-000463	32.51	32.91	−0.4	down	

miR-Name AB-Assay ID	Cq-Value Responder (R)	Cq-Value Non-Responder	Delta Cq R-(non-R)	miR-Regulation (Non-R to R) One-Sided with Cq<33	Assays Run on LC480
hsa-miR-202-002363	32.26	32.66	−0.4	down	
hsa-miR-20a-000580	25.35	25.75	−0.4	down	
hsa-let-7d-002283	29.94	30.35	−0.4	down	
hsa-miR-320-002277	26.40	26.80	−0.4	down	
hsa-miR-25-000403	30.51	30.93	−0.4	down	
hsa-miR-1290-002863	30.44	30.85	−0.4	down	
hsa-miR-196b-002215	27.55	27.97	−0.4	down	
hsa-miR-193b-002367	27.99	28.42	−0.4	down	
hsa-miR-16-000391	23.48	23.91	−0.4	down	
hsa-miR-571-001613	30.48	30.91	−0.4	down	
hsa-miR-339-3p-002184	29.81	30.26	−0.5	down	
hsa-miR-18a-002422	31.99	32.45	−0.5	down	
hsa-miR-1260-002896	25.63	26.10	−0.5	down	
hsa-miR-650-001603	29.40	29.87	−0.5	down	
hsa-miR-340-002258	31.94	32.42	−0.5	down	
hsa-miR-663B-002857	25.50	25.99	−0.5	down	
hsa-miR-215-000518	28.34	28.84	−0.5	down	
hsa-miR-337-5p-002156	33.29	33.79	−0.5	down	
hsa-miR-195-000494	25.52	26.03	−0.5	down	
hsa-miR-324-5p-000539	31.46	31.98	−0.5	down	
hsa-miR-501-001047	30.27	30.79	−0.5	down	
hsa-miR-1274B-002884	19.34	19.87	−0.5	down	
hsa-miR-15a#-002419	32.37	32.90	−0.5	down	
hsa-miR-99a-000435	26.41	26.96	−0.5	down	
hsa-miR-29a-002112	22.82	23.38	−0.6	down	
hsa-miR-31#-002113	29.58	30.14	−0.6	down	
hsa-miR-132-000457	28.52	29.09	−0.6	down	
hsa-miR-21#-002438	28.69	29.27	−0.6	down	
hsa-miR-598-001988	32.58	33.17	−0.6	down	
hsa-miR-101-002253	29.93	30.52	−0.6	down	
hsa-miR-361-000554	32.39	32.99	−0.6	down	
hsa-miR-649-001602	33.77	34.38	−0.6	down	
hsa-miR-708-002341	30.10	30.73	−0.6	down	
hsa-miR-130a-000454	29.21	29.84	−0.6	down	
hsa-miR-429-001024	29.63	30.27	−0.6	down	
hsa-miR-330-5p-002230	32.73	33.37	−0.6	down	
hsa-miR-296-000527	31.83	32.47	−0.6	down	
hsa-miR-10b-002218	27.52	28.16	−0.6	down	
hsa-miR-30b-000602	26.17	26.83	−0.7	down	
hsa-miR-1271-002779	31.37	32.03	−0.7	down	
hsa-miR-590-3P-002677	30.51	31.17	−0.7	down	
hsa-miR-139-5p-002289	30.29	30.96	−0.7	down	
hsa-miR-213-000516	30.95	31.62	−0.7	down	
hsa-miR-100-000437	26.41	27.08	−0.7	down	
hsa-miR-194-000493	26.33	27.01	−0.7	down	
hsa-miR-181a-000480	26.97	27.65	−0.7	down	
hsa-miR-29b-000413	29.34	30.02	−0.7	down	
hsa-miR-875-5p-002203	31.32	32.00	−0.7	down	
hsa-miR-192-000491	25.65	26.33	−0.7	down	
hsa-miR-484-001821	26.34	27.04	−0.7	down	

miR-Name AB-Assay ID	Cq-Value Responder (R)	Cq-Value Non-Responder	Delta Cq R-(non-R)	miR-Regulation (Non-R to R) One-Sided with Cq<33	Assays Run on LC480
hsa-miR-26b-000407	26.92	27.63	−0.7	down	
hsa-miR-27a-000408	27.30	28.04	−0.7	down	
hsa-miR-92a-000431	29.57	30.31	−0.7	down	
hsa-miR-32-002109	32.34	33.09	−0.8	down	
hsa-miR-455-001280	28.46	29.22	−0.8	down	
hsa-miR-720-002895	19.54	20.30	−0.8	down	
hsa-miR-374-000563	28.42	29.22	−0.8	down	
hsa-miR-1180-002847	31.34	32.16	−0.8	down	
hsa-let-7f-000382	30.10	30.92	−0.8	down	
hsa-miR-10b#-002315	30.07	30.89	−0.8	down	
hsa-miR-30d-000420	25.70	26.54	−0.8	down	
hsa-miR-886-5p-002193	26.58	27.42	−0.8	down	
hsa-miR-138-002284	31.57	32.45	−0.9	down	
hsa-miR-455-3p-002244	32.18	33.08	−0.9	down	
hsa-miR-542-5p-002240	32.42	33.31	−0.9	down	
hsa-miR-151-5P-002642	29.93	30.83	−0.9	down	
hsa-miR-1825-002907	28.43	29.33	−0.9	down	
hsa-miR-30e-3p-000422	26.18	27.09	−0.9	down	
hsa-miR-1244-002791	31.87	32.78	−0.9	down	
hsa-miR-186-002285	27.44	28.37	−0.9	down	
hsa-miR-595-001987	33.54	34.48	−0.9	down	
hsa-miR-30c-000419	25.27	26.21	−0.9	down	
hsa-miR-361-3p-002116	31.38	32.35	−1.0	down	
hsa-miR-93#-002139	30.20	31.17	−1.0	down	
hsa-miR-30d#-002305	31.10	32.08	−1.0	down	
hsa-miR-362-3p-002117	32.35	33.33	−1.0	down	
hsa-miR-210-000512	24.46	25.44	−1.0	down	
hsa-miR-339-5p-002257	30.09	31.08	−1.0	down	
hsa-miR-27b-000409	28.96	29.95	−1.0	down	
hsa-miR-886-3p-002194	25.46	26.48	−1.0	down	
hsa-miR-550-001544	31.18	32.21	−1.0	down	
hsa-miR-628-3p-002434	33.81	34.84	−1.0	down	
hsa-miR-133b-002247	33.11	34.14	−1.0	down	
hsa-miR-149-002255	30.43	31.48	−1.0	down	
hsa-miR-639-001583	31.34	32.39	−1.0	down	
hsa-miR-567-001534	33.92	34.97	−1.0	down	
hsa-miR-657-001512	33.01	34.08	−1.1	down	
hsa-miR-660-001515	26.90	27.98	−1.1	down	
hsa-miR-181c-000482	31.79	32.88	−1.1	down	
hsa-miR-135a-000460	30.25	31.36	−1.1	down	
hsa-miR-331-5p-002233	32.59	33.72	−1.1	down	
hsa-miR-532-001518	28.71	29.85	−1.1	down	
hsa-miR-378-002243	27.07	28.22	−1.1	down	
hsa-miR-30a-5p-000417	24.24	25.40	−1.2	down	
hsa-miR-106b#-002380	32.85	34.02	−1.2	down	
hsa-miR-362-001273	31.11	32.33	−1.2	down	
hsa-miR-148b#-002160	32.97	34.21	−1.2	down	
hsa-miR-26a-000405	25.17	26.43	−1.3	down	
hsa-miR-27b#-002174	32.96	34.25	−1.3	down	
hsa-miR-29c-000587	27.12	28.41	−1.3	down	

**miR-Name AB-Assay ID**	**Cq-Value Responder (R)**	**Cq-Value Non-Responder**	**Delta Cq R-(non-R)**	**miR-Regulation (Non-R to R) One-Sided with Cq<33**	**Assays Run on LC480**
mean RNU48-001006	25.50	26.84	−1.3	down	
hsa-miR-30a-3p-000416	25.15	26.54	−1.4	down	
hsa-miR-148b-000471	31.77	33.17	−1.4	down	
hsa-miR-92b#-002343	32.80	34.20	−1.4	down	
hsa-miR-345-002186	29.36	30.77	−1.4	down	
hsa-miR-146a-000468	25.71	27.12	−1.4	down	
hsa-miR-23a-000399	31.07	32.48	−1.4	down	
hsa-miR-23b-000400	30.95	32.37	−1.4	down	
hsa-miR-127-000452	31.82	33.25	−1.4	down	
hsa-miR-221-000524	28.54	29.97	−1.4	down	
hsa-miR-629-001562	33.70	35.16	**−1.5**	down	-
hsa-miR-1208-002880	32.31	33.78	**−1.5**	down	-
hsa-miR-625#-002432	30.58	32.04	**−1.5**	down	-
hsa-miR-744-002324	29.83	31.29	**−1.5**	down	-
hsa-miR-450a-002303	32.82	34.30	**−1.5**	down	-
hsa-miR-222-002276	25.20	26.69	**−1.5**	down	-
hsa-miR-99a#-002141	31.63	33.17	**−1.5**	down	-
hsa-miR-422a-002297	33.57	35.17	**−1.6**	down	-
hsa-miR-197-000497	29.48	31.10	**−1.6**	down	-
hsa-miR-26a-1#-002443	33.83	35.45	**−1.6**	down	-
hsa-miR-452-002329	29.90	31.58	**−1.7**	down	-
hsa-miR-622-001553	31.48	33.22	**−1.7**	down	-
hsa-miR-520D-3P-002743	33.79	35.54	**−1.8**	down	-
hsa-miR-597-001551	33.53	35.29	**−1.8**	down	-
hsa-miR-17#-002421	32.89	34.66	**−1.8**	down	-
hsa-miR-342-3p-002260	26.55	28.38	**−1.8**	down	**X**
hsa-miR-190-000489	32.37	34.23	**−1.9**	down	-
hsa-miR-675-002005	31.31	33.19	**−1.9**	down	-
hsa-miR-222#-002097	31.48	33.39	**−1.9**	down	-
hsa-miR-204-000508	26.24	28.16	**−1.9**	down	**X**
hsa-miR-500-002428	31.20	33.15	**−1.9**	down	**X**
hsa-miR-580-001621	33.09	35.04	**−2.0**	down	-
hsa-miR-486-3p-002093	31.35	33.33	**−2.0**	down	-
hsa-miR-200a#-001011	32.88	34.90	**−2.0**	down	-
hsa-miR-548d-5p-002237	32.92	35.07	**−2.1**	down	-
hsa-miR-1305-002867	32.79	34.96	**−2.2**	down	-
hsa-miR-296-3p-002101	33.66	35.86	**−2.2**	down	-
hsa-miR-545-002267	33.08	35.44	**−2.4**	down	-
hsa-miR-564-001531	32.35	34.81	**−2.5**	down	-
hsa-miR-20b-001014	28.44	30.93	**−2.5**	down	**X**
hsa-miR-203-000507	31.14	33.78	**−2.6**	down	**X**
hsa-miR-181c#-002333	31.43	34.14	**−2.7**	down	-
hsa-miR-1269-002789	31.45	34.25	**−2.8**	down	**X**
hsa-miR-644-001596	33.26	36.19	**−2.9**	down	-
hsa-miR-489-002358	27.08	30.58	**−3.5**	down	**X**

Information S2: RT-qPCR and droplet digital PCR methodologies.

General comments regarding the PCR guidelines and RNA quality data.

RT-qPCR measurements were performed according to the recommendations of the MIQE guidelines [1]. Corresponding comments are listed in the following checklist (Supplementary Table S2 and the Section "Pre-testing step: RT-qPCR with the LightCycler 480"). This also applies for the droplet digital PCR measurements according to the digital MIQE guidelines [2] shown in Supplementary Table S3 with corresponding comments and experimental details in the section "Validation step: Droplet digital PCR using the QX200 digital PCR instrument". No template controls (NTC) and no reverse-transcription controls (NRTC or no enzyme controls = NEC) were always performed and showed negative results.

RNA was extracted from formalin-fixed, paraffin-embedded (FFPE) tissue samples (mean: 15.5 ± 4.6 mg). The median RNA yield from one mg of FFPE tissue sample amounted to 961 ng (95% CI, 529–1746 ng) for non-responders and 1217 ng (95% CI, 691–1859) for responders and did not differ between the two cohorts (Mann–Whitney U-test, P = 0.499). The purity of isolated RNA samples defined by the 260 nm to 280 nm absorbance ratio (median, 95% CI 1.89, 1.84–1.92 for non-responders and 1.90, 1.85–1.93 for responders) also did not differ between the study groups (Mann–Whitney U-test, P = 0.669) and fulfilled the purity criterion for down-stream measurements. RNA integrity was not determined since our previous studies proved that miRNA stability is not decreased in total RNA samples with low RNA integrity [3].

Table S2. MIQE checklist according to Bustin et al. [1].

Item to Check	Importance	Checklist	Where?; Comment
**Experimental Design**			
Definition of experimental and control groups	E	Yes	Main text: Materials and Methods; Table I; Figure 1: Flow diagram
Number within each group	E	Yes	Main text: Materials and Methods; Table I; Figure 1: Flow diagram
Assay carried out by core lab or investigator's lab?	D	Yes	Investigator's lab
Acknowledgement of authors' contributions	D	No	
**SAMPLE**			
Description	E	Yes	Main text: Materials and Methods, Patients and samples; RNA extraction...; Results: Study design, patients and sample characteristics; Supplementary Information S2, General comments
Volume/mass of sample processed	D	Yes	Main text: Materials and Methods, Patients and samples; RNA extraction...; Results: Study design, patients and sample characteristics; Supplementary Information S2, General comments
Microdissection or macrodissection	E	Yes	Main text: Materials and Methods, Patients and samples; RNA extraction...; Results: Study design, patients and sample characteristics; Supplementary Information S2, General comments
Processing procedure	E	Yes	Main text: Materials and Methods, Patients and samples
If frozen - how and how quickly?	E	Not applicable	FFPE material; see below
If fixed - with what, how quickly?	E	Yes	Tissue fixation immediately after nephrectomy in neutral-buffered formaldehyde solution for tissue fixation
Sample storage conditions and duration (esp. for FFPE samples)	E	Yes	Main text: Materials and Methods: Patients and samples
**Nucleic Acid Extraction**			
Procedure and/or instrumentation	E	Yes	Main text: Materials and Methods, RNA extraction
Name of kit and details of any modifications	E	Yes	Main text: Materials and Methods, RNA extraction: miRNeasy FFPE Kit (Qiagen, Cat. No. 217504)
Source of additional reagents used	D	No	

Details of DNase or RNAse treatment	E	Yes	Main text: Materials and Methods, RNA extraction: on-column DNase digestion
Contamination assessment (DNA or RNA)	E	Yes	Main text: Materials and Methods, RNA extraction: on-column DNase digestion; Supplementary Information S2: General comments: DNA contamination was excluded by reaction without reverse transcription in pilot experiments; in addition, this principle of miRNAs is not affected by genomic DNA contamination according to the manufacturer
Nucleic acid quantification	E	Yes	Main text: Materials and Methods; RNA extraction: NanoDrop ND-1000; spectrophotometry
Instrument and method	E	Yes	Main text: Materials and Methods; RNA extraction: NanoDrop ND-1000; spectrophotometry
Purity (A260/A280)	D	Yes	Main text: Results, Study design and characteristics of patients and samples; Supplementary Information S2, General comments
Yield	D	Yes	Main text: Results, Study design and characteristics of patients and samples; Supplementary Information S2, General comments
RNA integrity method/instrument	E	Yes	Main text: Results, Study design and characteristics of patients and samples; Supplementary Information S2: General comments
RIN/RQI or Cq of 3' and 5' transcripts	E	Yes	Supplementary Information S2: General comments
Electrophoresis traces	D	No	
Inhibition testing (Cq dilutions, spike or other)	E	Yes	Supplementary Information S2 with Supplementary Figure. S1 with standard curves and their characteristics
Storage conditions (Nucleic acid): temperature, concentration, duration, buffer)	E	Yes	Main text: RNA extraction...: storage in nuclease-free water in two aliquots after isolation at −80 °C up to cDNA synthesis
**Reverse Transcription**			
Complete reaction conditions	E	Yes	Supplementary Information S2: cDNA synthesis and RT-PCR methodology
Amount of RNA and reaction volume	E	Yes	Supplementary Information S2: cDNA synthesis
Priming oligonucleotide (if using GSP) and concentration	E	Yes	Supplementary Information S2: cDNA synthesis, Supplementary Table S4
Reverse transcriptase and concentration	E	Yes	Supplementary Information S2: cDNA synthesis
Temperature and time	E	Yes	Supplementary Information S2: cDNA synthesis a
Manufacturer of reagents and catalogue numbers	D	Yes	Main text: Materials and Methods, ...miRNA quantification and Supplementary Information S2: cDNA synthesis
Cqs with and without RT	D	Yes	Supplementary Information S2: RT-PCR methodology, General comments; see also comment on DNase treatment concerning miRNAs
Storage conditions of cDNA	D	Yes	Supplementary Information S2: cDNA synthesis; storage at −20 °C
**qPCR Target Information**			
Gene symbols	E	Yes	Main text: Materials and Methods, ...miRNA quantification and Supplementary Information S2: Supplementary Table S4
If multiplex, efficiency and LOD of each assay.	E	Not applicable	
Sequence accession number	E	Yes	Main text: Materials and Methods, ...miRNA quantification and Supplementary Information S2: Supplementary Table S4
Location of amplicon	D	Yes	Supplementary Information S2: Supplementary Table S4; controlled by the manufacturer of the miRNA assays (Thermo Fisher, Applied Biosystems)
Amplicon length	E	Yes	Controlled by the manufacturer of the miRNA assays (Thermo Fisher, Applied Biosystems); own electrophoretic results showed 60–80 bp

In silico specificity screen (BLAST, etc.)	E	Yes	Controlled by the manufacturer of the miRNA assays (Thermo Fisher, Applied Biosystems)
Pseudogenes, retropseudogenes or other homologs?	D	Yes	Controlled by the manufacturer of the miRNA assays (Thermo Fisher, Applied Biosystems)
Sequence alignment	D	Yes	Controlled by the manufacturer of the miRNA assays (Thermo Fisher, Applied Biosystems)
Secondary structure analysis of amplicon	D	Yes	Controlled by the manufacturer of the miRNA assays (Thermo Fisher, Applied Biosystems)
Location of each primer by exon or intron (if applicable)	E	Yes	Controlled by the manufacturer of the miRNA assays (Thermo Fisher, Applied Biosystems)
What splice variants are targeted?	E	Yes	Controlled by the manufacturer of the miRNA assays (Thermo Fisher, Applied Biosystems)
**qPCR Oligonucleotides**			
Primer sequences	E	Yes	Supplementary Information S2, quantitative real-time PCR: use of commercial kits (Thermo Fisher, Applied Biosystems); Supplementary Table S4
RTPrimerDB Identification Number	D	No	
Probe sequences	D	Yes	Supplementary Information S2, quantitative real-time PCR: use of commercial kits (Thermo Fisher, Applied Biosystems). Manufacturer does not provide this information
Location and identity of any modifications	E	Yes	Supplementary Information S2, quantitative real-time PCR: use of commercial kits (Thermo Fisher, Applied Biosystems), Supplementary Table S4
Manufacturer of oligonucleotides	D	Yes	Supplementary Information S2, quantitative real-time PCR: use of commercial kits (Thermo Fisher, Applied Biosystems). Manufacturer does not provide this information
Purification method	D	Not applicable	Supplementary Information S2, quantitative real-time PCR: use of commercial kits (Thermo Fisher, Applied Biosystems). Manufacturer does not provide this information
**qPCR Protocol**			
Complete reaction conditions	E	Yes	Main text: Materials and Methods:.....miRNA quantification and Supplementary Information S2, using commercial tests, Supplementary Table S4 and quantitative real-time PCR
Reaction volume and amount of cDNA/DNA	E	Yes	Main text: Materials and Methods, ...miRNA quantification; Supplementary Information S2, quantitative real-time PCR
Primer, (probe), Mg++ and dNTP concentrations	E	Yes	Main text: Materials and Methods, ...miRNA quantification; Supplementary Information S2, quantitative real-time PCR; details not provided by the manufacturer
Polymerase identity and concentration	E	Yes	Main text: Materials and Methods, ...miRNA quantification; Supplementary Information S2 and Supplementary Table S4, quantitative real-time PCR; details not provided by the manufacturer
Buffer/kit identity and manufacturer	E	Yes	Main text: Materials and Methods, ...miRNA quantification; Supplementary Information S2 and Supplementary Table S4, quantitative real-time PCR; details not provided by the manufacturer
Exact chemical constitution of the buffer	D	No	Use of commercial tests, Supplementary Table S4; details are not provided by the manufacturer
Additives (SYBR Green I, DMSO, etc.)	E	Not applicable	
Manufacturer of plates/tubes and catalogue number	D	Yes	Main text: Materials and Methods, ..miRNA quantification; Supplementary Information S2: quantitative real-time PCR white 96-well plates (Roche)
Complete thermocycling parameters	E	Yes	Supplementary Information S2: quantitative real-time PCR
Reaction setup (manual/robotic)	D	Yes	Manual setup

Manufacturer of qPCR instrument	E	Yes	Main text: Materials and Methods, ...miRNA quantification; Supplementary Information S2: quantitative real-time PCR (LightCycler, Roche)
**qPCR Validation**			
Evidence of optimisation (from gradients)	D	No	Main text: Materials and Methods, ...miRNA quantification; Supplementary Information S2,: quantitative real-time PCR: Supplementary Table S4, use of commercial kits (Thermo Fisher, Applied Biosystems)
Specificity (gel, sequence, melt, or digest)	E	Yes	Controlled by the manufacturer of the miRNA assays (Thermo Fisher, Applied Biosystems)
For SYBR Green I, Cq of the NTC	E	Not applicable	
Standard curves with slope and y-intercept	E	Yes	Supplementary Information S2: Performance data of qPCR with Supplementary Figure S1 and legends
PCR efficiency calculated from slope	E	Yes	Supplementary Information S2: Performance data of qPCR with Supplementary Figure S1 and legends
Confidence interval for PCR efficiency or standard error	D	Yes	Supplementary Information S2: Performance data of qPCR with Supplementary Figure S1 and legends
r2 of standard curve	E	No	Not provided by the LC480 software
Linear dynamic range	E	Yes	Supplementary Information S2: quantitative real-time PCR
Cq variation at lower limit	E	Yes	Supplementary Information S2: Performance data with Supplementary Table S5
Confidence intervals throughout range	D	No	
Evidence for limit of detection	E	No	Dynamic range from 11 to 35 Cqs, but all measured miRNAs had Cqs <34
If multiplex, efficiency and LOD of each assay.	E	Not applicable	
**Data Analysis**			
qPCR analysis program (source, version)	E	Yes	Main text: Materials and Methods, ..miRNA quantification; Supplementary Information S2: quantitative real-time PCR with LightCycler software, release 1.5.0 using the "second derivative maximum" method
Cq method determination	E	Yes	
Outlier identification and disposition	E	Not applicable	
Results of NTCs	E	Yes	Supplementary Information S2: General comments
Justification of number and choice of reference genes	E	Yes	Main text: Materials and Methods, ...miRNA quantification; in the pretesting approach using LightCycler, miR-103 was initially envisaged as normalizer, but the validation approach was continued using droplet digital PCR without the necessity of normalizers
Description of normalisation method	E	Not applicable	See previous comment
Number and concordance of biological replicates	D	Yes	Example of concordance between matched samples measured on the array platform and LightCycler: Supplementary Figure S2
Number and stage (RT or qPCR) of technical replicates	E	Yes	Supplementary Information S2: ...miRNA quantification using triplicates. Example of concordance between matched samples measured on the array platform and LightCycler: Supplementary Figure S2
Repeatability (intra-assay variation)	E	Yes	See reproducibility data as preferred performance criterion
Reproducibility (inter-assay variation, %CV)	D	Yes	Supplementary Information S2: Performance data, see Supplementary Table S5
Power analysis	D	Yes	Main text: Results: Study design, patients and sample characteristics
Statistical methods for result significance	E	Yes	Main text: Materials and Methods, Data analysis and statistics
Software (source, version)	E	Yes	Main text: Materials and Methods: Data analysis and statistics
Cq or raw data submission using RDML	D	No	

Table S3. Digital MIQE checklist according to Huggett et al. [2].

Item to Check	Importance	Checklist	Where?; Comment
**Experimental Design**			
Definition of experimental and control groups	E	Yes	Main text: Materials and Methods; Table I; Figure 1: Flow diagram
Number within each group	E	Yes	Main text: Materials and Methods; Table I; Figure 1: Flow diagram
Assay carried out by core lab or investigator's lab?	D	Yes	Investigator's lab
Power analysis	D	Yes	Main text: Results: Study design, patients and sample characteristics
**Sample**			
Description	E	Yes	Main text: Materials and Methods, Patients and samples; RNA extraction...; Results: Study design, patients and sample characteristics; Supplementary Information S2, General comments
Volume/mass of sample processed	D	Yes	Main text: Materials and Methods, Patients and samples; RNA extraction...; Results: Study design, patients and sample characteristics; Supplementary Information S2, General comments
Microdissection or macrodissection	E	Yes	Main text: Materials and Methods, Patients and samples; RNA extraction...; Results: Study design, patients and sample characteristics; Supplementary Information S2, General comments
Processing procedure	E	Yes	Main text: Materials and Methods, Patients and samples
If frozen - how and how quickly?	E	Not applicable	FFPE material; see below
If fixed - with what, how quickly?	E	Yes	Tissue fixation immediately after nephrectomy in neutral-buffered formaldehyde solution for tissue fixation
Sample storage conditions and duration (especially for FFPE samples)	E	Yes	Main text: Materials and Methods: Patients and samples
**Nucleic Acid Extraction**			
Manufacturer of reagents used and catalogue number	D	Yes	Main text: Materials and Methods, RNA extraction: miRNeasy FFPE Kit (Qiagen, Cat. No. 217504)
Quantification -instrument/method	E	Yes	Main text: Materials and Methods; RNA extraction: NanoDrop ND-1000; spectrophotometry
DNA or RNA quantification	E	Yes	RNA
Quality/Integrity, method/instrument, e.g. RNA integrity	E	Yes	Supplementary Information S2: General comments: A260/A280 ratio; Main text, Results: Study design, patients and sample characteristics
Template structural information	E	Yes	See above; RNA extraction using miRNeasy FFPE Kit Qiagen
Template modification (digestion, sonication, preamplification, etc.)	E	Yes	Main text: Materials and Methods, RNA extraction. : deparaffinization, tissue lysis (TissueLyser, Qiagen), proteinase K digestion
Template treatment	E	Yes	Main text: Materials and Methods, RNA extraction. :deparaffinization, tissue lysis (TissueLyser, Qiagen), proteinase K digestion
Inhibition dilutions or spike	E	Yes	See Supplementary Table S2 and standard curves in Supplementary Figure S1; cDNA was diluted according to dilution schemes indicated in Supplementary Table S6
DNA contamination assessment of RNA samples	E	Yes	Main text: Materials and Methods, RNA extraction: on-column DNase digestion; Supplementary Information S2: General comments: DNA contamination was excluded by reaction without reverse transcription in pilot experiments; in addition, this principle of miRNAs is not affected by genomic DNA contamination according to the manufacturer

Details of DNase treatment where performed	E	Yes	See previous comment: on-column DNase treatment
Storage conditions (Nucleic acid): temperature, concentration, duration, buffer)	E	Yes	Main text: RNA extraction...: storage in nucleases-free water in two aliquots after isolation at −80 °C up to cDNA synthesis
**Reverse Transcription (if necessary)**			
cDNA priming method and concentration	E	Yes	Main text: Materials and Methods, ...miRNA quantification; Supplementary Information S2, cDNA synthesis-specific stem-loop RT primer, see Supplementary Table S4
One- or two-step protocol	E	Yes	Main text: Materials and Methods, two-step protocol
Amount of RNA used per reaction	E	Yes	Supplementary Information S2: cDNA synthesis
Detailed reaction components and conditions	E	Yes	Supplementary Information S2: cDNA synthesis
RT efficiency	D	No	
Estimated copies measured with and without addition of RT	D	Yes	Supplementary Information S2, General comments
Manufacturer of reagents and catalogue numbers	D	Yes	Main text: Materials and Methods, ...miRNA quantification and Supplementary Information S2: cDNA synthesis
Reaction volume	D		Supplementary Information S2: cDNA synthesis
Storage conditions of cDNA	D		Supplementary Information S2: cDNA synthesis; storage at −20 °C
**dPCR Target Information**			
Sequence accession number	E	Yes	Main text: Materials and Methods, ...miRNA quantification and Supplementary Information S2: Supplementary Table S4
Location of amplicon	D	Yes	Supplementary Information S2: Supplementary Table S4; controlled by the manufacturer of the miRNA assays (Thermo Fisher, Applied Biosystems)
Amplicon length	E	Yes	Controlled by the manufacturer of the miRNA assays (Thermo Fisher, Applied Biosystems); own electrophoretic results showed 60-80 bp
In silico specificity screen (BLAST, etc)	E	Yes	Controlled by the manufacturer of the miRNA assays (Thermo Fisher, Applied Biosystems)
Pseudogenes, retropseudogenes or other homologs?	D	Yes	Controlled by the manufacturer of the miRNA assays (Thermo Fisher, Applied Biosystems)
Sequence alignment	D	Yes	Controlled by the manufacturer of the miRNA assays (Thermo Fisher, Applied Biosystems)
Secondary structure analysis of amplicon and GC content	D	Yes	Controlled by the manufacturer of the miRNA assays (Thermo Fisher, Applied Biosystems)
Location of each primer by exon or intron (if applicable)	E	Yes	Controlled by the manufacturer of the miRNA assays (Thermo Fisher, Applied Biosystems)
What splice variants are targeted?	E	Yes	Controlled by the manufacturer of the miRNA assays (Thermo Fisher, Applied Biosystems)
**dPCR Oligonucleotides**			
Primer sequences	E	Yes	Supplementary Information S2, Supplementary Table S4: use of commercial kits (Thermo Fisher, Applied Biosystems)
RTPrimerDB Identification Number	D	No	
Probe sequences	D	Yes	Main text: Materials and Methods...miRNA quantification: use of commercial kits (Thermo Fisher, Applied Biosystems). Manufacturer does not provide this information
Location and identity of any modifications	E	Yes	Main text: Materials and Methods...miRNA quantification: use of commercial kits (Thermo Fisher, Applied Biosystems), Supplementary Table S4
Manufacturer of oligonucleotides	D	Yes	Main text: Materials and Methods...miRNA quantification: use of commercial kits (Thermo Fisher, Applied Biosystems). Manufacturer does not provide this information
Purification method	D	Not applicable	Main text: Materials and Methods...miRNA quantification: use of commercial kits (Thermo

			Fisher, Applied Biosystems). Manufacturer does not provide this information
**dPCR Protocol**			
Complete reaction conditions	E	Yesq	Main text: Materials and Methods, miRNA quantification; Supplementary Information S2, Supplementary Table S6
Reaction volume and amount of cDNA/DNA	E	Yes	Main text: Materials and Methods, miRNA quantification; Supplementary Information S2, Supplementary Table S6
Primer, (probe), Mg++ and dNTP concentrations	E	Yes	Main text: Materials and Methods, miRNA quantification; Supplementary Information S2, Supplementary Tables S4 and 6; not all details are provided by the manufacturer
Polymerase identity and concentration	E	Yes	Main text: Materials and Methods, miRNA quantification; Supplementary Information S2, Supplementary Tables S4 and 6; not all details are provided by the manufacturer
Buffer/kit identity and manufacturer	E	Yes	Main text: Materials and Methods, miRNA quantification; Supplementary Information S2, Supplementary Tables S4 and 6
Exact chemical constitution of the buffer	D	No	Main text: Materials and Methods, miRNA quantification; Supplementary Information S2, Supplementary Tables S4 and 6, but not all details are provided by the manufacturer
Additives (SYBR Green I, DMSO, etc.)	E	Not applicable	
Plates/tubes catalogue number and manufacturer	D	Yes	Main text: Materials and Methods, miRNA quantification; Supplementary Information S2, Supplementary Table S6
Complete thermocycling parameters	E	Yes	Main text: Materials and Methods, miRNA quantification; Supplementary Information S2, Supplementary Table S6
Reaction setup (manual/robotic)	D	Yes	Manual setup
Gravimetric or volumetric dilutions (manual/robotic)	D	Yes	Supplementary Information S2, Supplementary Table S6
Total PCR volume prepared	D	Yes	Main text: Materials and Methods, miRNA quantification; Supplementary Information S2, Supplementary Table S6
Partition number	E	Yes	Supplementary Information S2, Supplementary Tables S6–8, Supplementary Figure S3
Individual partition volume	E	Yes	Provided by the manufacturer: 1 nL
Total volume of the partitions measured (effective reaction size)	E	Yes	Supplementary Information S2, Supplementary Tables S6–8, Supplementary Figure S3
Partition volume variance/SD	D	Yes	Supplementary Information S2, Supplementary Tables S6–8, Supplementary Figure S3
Comprehensive details and appropriate use of controls	E	Yes	Supplementary Information S2, Supplementary Table S8
Manufacturer of dPCR instrument	E	Yes	Main text: Materials and Methods, miRNA quantification; Supplementary Table S6 (QX200 ddPCR System from BIO-RAD)
**dPCR Validation**			
Optimisation data for the assay	D	Yes	Only partly optimized by optimizing the annealing temperature using thermal gradients since the original tests provided reliable results as shown in control experiments (Supplementary Table S8)
Specificity (when measuring rare mutations, pathogen sequences, etc.)	E	Not applicable	
Limit of detection of calibration control	D	Not applicable	
If multiplexing, comparison with singleplex assays	E	Not applicable	
**DATA Analysis**			
Mean copies per partition (λ or equivalent)	E		Supplementary Information S2, Supplementary Table S7

dPCR analysis program (source, version)	E	Yes	Main text: Materials and Methods, miRNA quantification; Supplementary Information S2, Supplementary Table S6: QuantaSoft Software Vs. 1.7.4.0917 from BIO-RAD
Outlier identification and disposition	E	Yes	Supplementary Information S2, Supplementary Table S3: legend
Results of NTCs	E	Yes	Supplementary Information S2, General comments
Examples of positive(s) and negative experimental results as supplemental data	E	No	
Where appropriate, justification of number and choice of reference genes	E	No	Main text: Materials and Methods, ...miRNA quantification; in the pretesting approach using LightCycler, miR-103 was initially envisaged as normalizer, but the validation approach was continued using droplet digital PCR without the necessity of normalizers
Where appropriate, description of normalization method	E	No	
Number and concordance of biological replicates	D	Yes	Example of concordance between matched samples measured on the array platform and on LightCycler in the pretesting step: Supplementary Figure S2. Independent biological replicates as shown in the study were preferred
Number and stage (RT or qPCR) of technical replicates	E	Yes	Duplicate qPCR measurements, see Supplementary Table S8, Part A
Repeatability (intra-assay variation)	E	Yes	Supplementary Information S2, Supplementary Table S8, Part A
Reproducibility (inter-assay/user/lab etc variation)	D	Yes	Supplementary Information S2, Supplementary Table S8, Part B
Experimental variance or CI d	E	Yes	Supplementary Information S2, Supplementary Table S8
Statistical methods for analysis	E	Yes	Main text: Materials and Methods, Data analysis and statistics
Data submission using RDML (Real-time PCR Data Markup Language)	D	No	

In the following sections, the quantification of the selected differentially expressed miRs and analytical performance data for all the measurements are compiled.

Pre-testing step: RT-qPCR analyses with the LightCycler 480.

The 11 selected differentially expressed miRNAs using the card array technique were measured in the pretesting step using TaqMan microRNA Reverse-Transcription Kits (Supplementary Table S4) with details given in the following and based on our previous publications [3–6].

Table S4. TaqMan MicroRNA assays used for RT-qPCR and droplet digital PCR: nomenclature and sequences of the miRNAs. Assay names and assay IDs are taken from the nomenclature of the supplier Thermo Fisher. The miRBase accession no., miRBase ID, and the target sequences were taken from the miRBase database release 22 (Sanger Institute, Manchester, UK; http://www.mirbase.org).

miRBase ID Release Version 22	miRBase Accession No.	AB Assay Name	AB Assay ID	Sequence	Chromosome Location/ Coordinates on Build GRCh38
hsa-miR-9-5p	MIMAT0000441	hsa-miR-9	000583	UCUUUGGUUAUCUAGCUGUAUGA	Chr. 1: 156420341 - 156420429 [−]
hsa-miR-20b-5p	MIMAT0001413	hsa-miR-20b	001014	CAAAGUGCUCAUAGUGCAGGUAG	Chr.X: 134169809 - 134169877 [−]
hsa-miR-203a-3p	MIMAT0000264	hsa-miR-203	000507	GUGAAAUGUUUAGGACCACUAG	Chr.14: 104117405 - 104117514 [+]
hsa-miR-204-5p	MIMAT0000265	hsa-miR-204	000508	UUCCCUUUGUCAUCCUAUGCCU	Chr. 9: 70809975 - 70810084 [−]

hsa-miR-223-3p	MIMAT0000280	hsa-miR-223	002295	UGUCAGUUUGUCAAAUACCCCA	Chr. X: 66018870 - 66018979 [+]
hsa-miR-342-3p	MIMAT0000753	hsa-miR-342-3p	002260	UCUCACACAGAAAUCGCACCCGU	Chr.14: 100109655 - 100109753 [+]
hsa-miR-483-5p	MIMAT0004761	hsa-miR-483-5p	002338	AAGACGGGAGGAAAGAAGGGAG	Chr.11: 2134134 - 2134209 [−]
hsa-miR-489-3p	MIMAT0002805	hsa-miR-489	002358	GUGACAUCACAUAUACGGCAGC	Chr. 7: 93483936 - 93484019 [−]
hsa-miR-500a-5p	MIMAT0004773	hsa-miR-500	002428	UAAUCCUUGCUACCUGGGUGAGA	Chr. X: 50008431 - 50008514 [+]
hsa-miR-885-5p	MIMAT0004947	hsa-miR-885-5p	002296	UCCAUUACACUACCCUGCCUCU	Chr. 3: 10394489 - 10394562 [−]
hsa-miR-1269a	MIMAT0005923	hsa-miR-1269	002789	CUGGACUGAGCCGUGCUACUGG	Chr.4: 66276824 - 66276928 [+]
&hsa-miR-103a-3p	MIMAT0000101	hsa-miR-103	000439	AGCAGCAUUGUACAGGGCUAUGA	Chr. 5: 168560896 - 168560973 [−]

&Hsa-miR-103a-3p was initially envisaged to be used as normalizer for the RT-qPCR analyses.

cDNA synthesis.

Total RNA was transcribed in cDNA with the TaqMan MicroRNA Reverse-Transcription Kit (Thermo Fisher, Applied Biosystems, Cat. No. 4366596) and miRNA specific stem-loop RT primers (Thermo Fisher, Applied Biosystems, Part No: 4427975). A final reaction mix of 10 µL included: 0.1 µL of dNTPs (100 mM total), 0.67 µL of MultiScribe RT (50 U/µL MuLV-RT), 1 µL of 10x RT-Buffer, 0.13 µL of RNase inhibitor (20 U/µL), 1 µL of miR-specific 5x RT primer, 1 µL of RNA (6.67 ng), and 6.1 µL of nuclease-free water. The transcription and detection of gDNA was separately controlled by full reaction mix without MultiScribe reverse transcriptase addition for each individual miRNA. The transcription was performed on a thermal block cycler with heated lid (Biometra, Göttingen, Germany) using the following conditions: 30 min at 16 °C (annealing), 30 min at 42 °C (transcription), 5 min at 85 °C (enzyme inactivation), final cooling to 4 °C and storage of cDNA at −20 °C until analysis.

Quantitative real-time PCR.

In the pretesting step, the individual TaqMan miRNA assays were performed on the LightCycler 480 Instrument (Roche Applied Science, Mannheim, Germany) in white 96-well plates (Roche, Cat. No. 04729692001) covered with instrument specific sealing foils. Each well contained 10 µL of following components: 5 µL of TaqMan 2x Universal PCR Master Mix No AmpErase UNG (Thermo Fisher, Applied Biosystems; PN 4324018), 3.5 µL of nuclease-free water, 0.5 µL of 20x miR-specific TaqMan MicroRNA Assay primer (Applied Biosystems, PN 4427975), and 1 µL of cDNA. The PCR run conditions were as follows: hot-start polymerase activation at 95 °C for 10 min, 45 amplification cycles with strand dissociation at 95 °C for 15 s, and annealing/elongation at 60 °C for 60 s with fluorescence acquisition, and a final cooling step at 40 °C for 60 s. The Cq values were extracted by the LightCycler 480 software v1.5 based on the "second derivative maximum" method and imported into a special data format in qBasePLUS Software v2.6 (Biogazelle, Zwijnaarde, Belgium) for statistical analysis. Three standard curves (miR-9-5p, miR-489-3p, and reference miR-103-3p) were generated with the LC480 software by 10-fold dilution steps over 7 logs of a 1:2 pre-diluted cDNA mixed with a 1:1000 pre-diluted amplicon. Because the efficiencies of all three standard curves ranged between 1.93 and 1.98, we recalculated a new standard curve with the input of all generated Cq values of measured three miRs. The overall standard curve had an efficiency of 1.95 (see the following section "Performance data of RT-qPCR analyses" with Figure S1). The linear dynamic range of the overall standard curve was between Cq 11.06 and Cq 35.24. All samples were in this measuring range. However, we defined a Cq value of 34.0 as lower limit for the quantification in the qBasePLUS software to achieve reliable expression data. The abovementioned qPCR efficiency value was used in qBasePLUS for the efficiency corrected calculation of the relative quantification.

Performance data of the RT-qPCR analyses

The performance data include the following figures and tables with the standard PCR curves (Supplementary Figure S1, reproducibility data (Supplementary Table S5), and correlation data between array data and RT-PCR results (Supplementary Figure 2).

miR-103a-3p

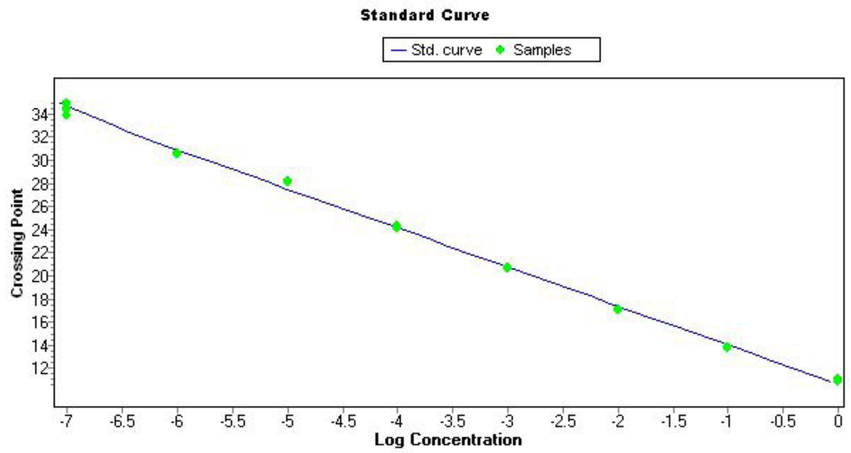

Error: 0.0330
Efficiency: 1.979
Slope: −3.373
y-Intercept: 10.61
Link: 0.000

miR-9-5p

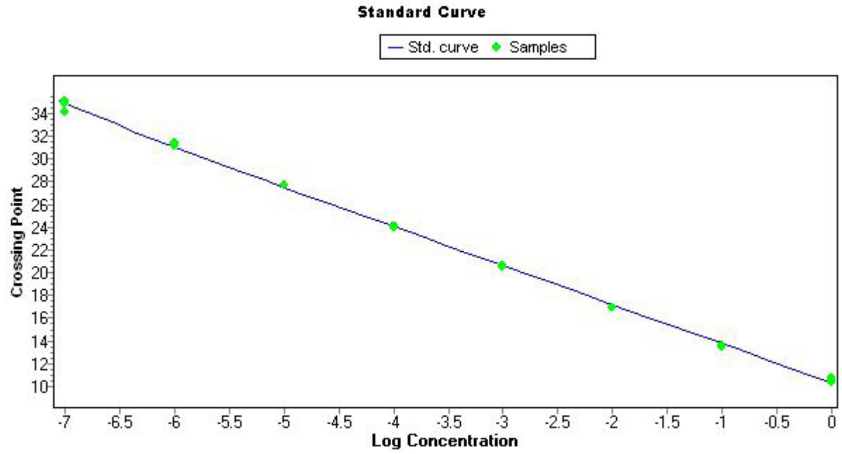

Error: 0.0195
Efficiency: 1.955
Slope: −3.434
y-Intercept: 10.22
Link: 0.00000880

miR-489-3p

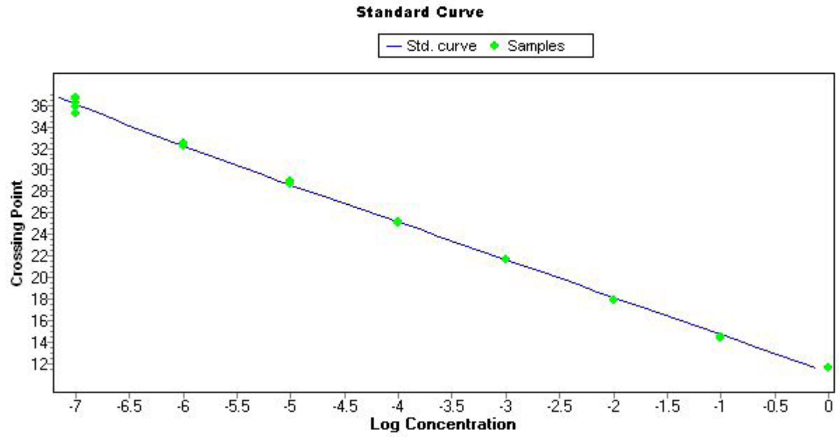

Error: 0.0214
Efficiency: 1.934
Slope: −3.491
y-Intercept: 11.07
Link: 0.00000877

all miRs

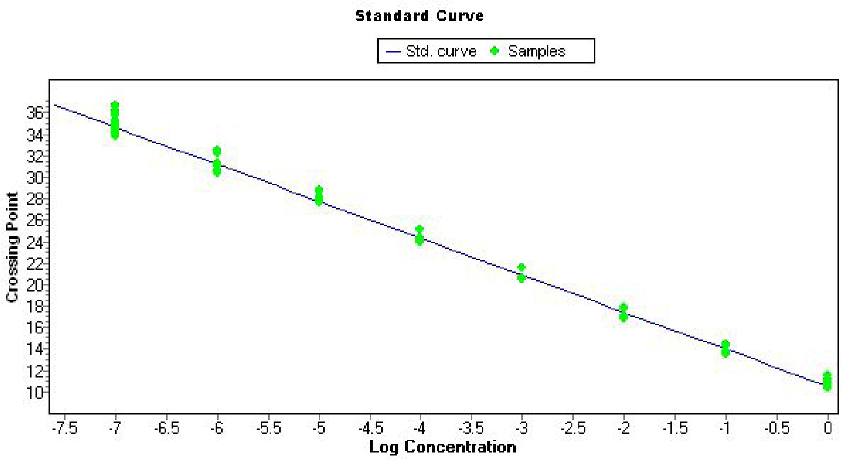

Error: 0.0282
Efficiency: 1.950
Slope: −3.447
y-Intercept: 10.50
Link: 0.000

Figure S1. Characteristics of the standard curves of qPCR analyses Standard curves were generated either from diluted cDNAs or from diluted amplicons. Cq values were calculated by the LightCycler Software Version 1.5.0 using the "second derivative maximum" method. The efficiency, the slope, intercept, and error of the regression line were calculated by the LightCycler 480 software. The PCR-efficiency is calculated by the LightCycler480 software after the formula: Efficiency=$10^{-1/slope}$. According to the LightCycler 480 operator's manual, the error value is the mean squared error of the single data points fit to the regression line.

Supplementary Table S5. Reproducibility of miRNA measurements.

**Hsa-miR**	**Concentrations**		**Replicates (n)**
	Mean (SD)	%RSD	
**miR-9-5p**			
level 1	9.995 (0.417)	4.18	4
level 2	0.049 (0.003)	7.07	5
**miR-489-3p**			
level 1	9.930 (0.377)	3.80	4
level 2	0.091 (0.006)	6.52	5

Abbreviation: %RSD = percent relative standard deviation.

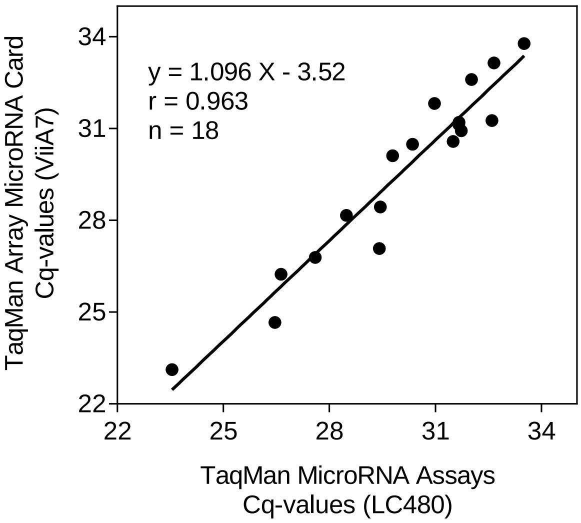

Supplementary Figure S2. Correlation between Array and RT-qPCR data of single miRNAs. The nine selected miRNAs examined in the validation phase showed strongly correlated Cq values in the two sample pools measured on the two platforms.

Validation step: Droplet digital PCR with the QX200 digital PCR instrument

Details of the cDNA synthesis are described above. In addition to the brief description given in the Materials and Methods of the main text, we summarized here further methodical details (Supplementary Table S6, Supplementary Figure 3, and Supplementary Table 7) and analytical performance data of the droplet digital PCR (Supplementary Table S8) as well as miRNA expression data (Supplementary Figure S4, Supplementary Table S9) in relation to clinicopathological factors in order to support the results presented in the main text.

Methodical details of the droplet digital PCR

Methodical details are given in the following Supplementary Tables S6 and S7 and in Supplementary Figure S3.

Table S6: Measurement conditions of droplet digital PCR analyses.

**Instrument**
QX200 ddPCR System (BIO-RAD, Germany, Munich) including following instruments (all from BIO-RAD):
QX200 Droplet Generator
PX1 PCR Plate Sealer
T100 Thermal Cycler
QX200 Droplet Reader
Reagents
2x ddPCR Supermix for Probes (no dUTP, BIO-RAD Cat. No. #186-3024)
miR-specific Primer/Probes (TaqMan microRNA assays, Thermo Fisher Scientific, Applied Biosystems; Supp. Table S4)

Experimental steps.

1.	Dispense 21 µL of ddPCR reaction mix (adjusted volume on pipet) up to the first hub of pipet (about 20 µL) without bubbles into the 8 sample-wells of the DG8 Cartridge (BIO-RAD Cat. No. #186-4008)
2.	Add 70 µL of Droplet Generator Oil for Probes (BIO-RAD Cat. No. #186-3005) into the 8 oil-well of a DG8 Cartridge (BIO-RAD Cat. No. #186-4008)
3.	Cover cartridge with Droplet Generator DG8 Gasket (BIO-RAD Cat. No. #186-3009) and place the full cartridge into QX200 Droplet Generator (BIO-RAD)
4.	Remove the cartridge from the Droplet Generator, remove the gasket and transfer 40 µL of droplets with a 8-channel pipet (Pipet-Lite XLS, 5-50 µL LTS Rainin, Mettler-Toledo, Germany) into one column of a ddPCR 96-well plate (BIO-RAD, Cat. No #12001925)
5.	Cover the 96-well plate with Pierceable Foil Heat Seal (BIO-RAD Cat. No. #181-4040) and insert the plate into heat block of the PX1 PCR plate sealer (BIO-RAD) and start the heat-sealing of 96-well plate with the foil for 5 s at 180°C
6.	Transfer the 96-well plate into the T100 Thermal Cycler (BIO-RAD) and start PCR run
7.	After finishing PCR, transfer of 96-well plate into the QX200 Droplete Reader (BIO-RAD)
8.	Read out of droplets per well with ddPCR Droplet Reader Oil (BIO-RAD Cat. No. #186-3004) and calculate copies of target miR/µL ddPCR reaction mix using QuantaSoft Software Version 1.7.4. (BIO-RAD)

Pipetting scheme for 1x Reaction mix/sample:

**Volume (µL)**	**Component**
11	2x ddPCR Supermix for Probes
1.1	20x miR-specific primer/probe
8.8	Nuclease free water
1.1	miR-specific cDNA (diluted or undiluted)
22	total volume of ddPCR reaction mix

miR dependend volumes of cDNA were added

**Target miRNA**	**µL cDNA (predilution)/22 µL ddPCR Reaction Mix**	**ddPCR Cycle Number**
hsa-miR-9-5p	2.2	45
hsa-miR-103a-3p	1	43

hsa-miR-203a-3p	1.1	40
hsa-miR-204-5p	1.1 (1:2)	40
hsa-miR-223-3p	1.1 (1:2)	40
hsa-miR-489-3p	2.2	45
hsa-miR-500a-5p	1.1	45
hsa-miR-885-5p	1.1	40
(total volume cDNA + H_2O = 9.9 µL per ddPCR mix)		

Thermal cycling steps for ddPCR run:

Instrument: T100 Thermal Cycler (Bio-Rad Laboratories GmbH, Germany, Munich); Ramp rate: 2 °C/s before and after annealing step (1 min); lid: 105 °C

Step	Time	Temperature (°C)
HOLD	10 min	95
39-44x {	30 s	94
	1 min	56
HOLD	10 min	98
HOLD	∞	4

Gene name miRBase v.22	AB assay ID	Fluorescence amplitudes of positive and negative droplets/assay	Mean number of accepted droplets/well	%R SD
miR-9-5p	000583	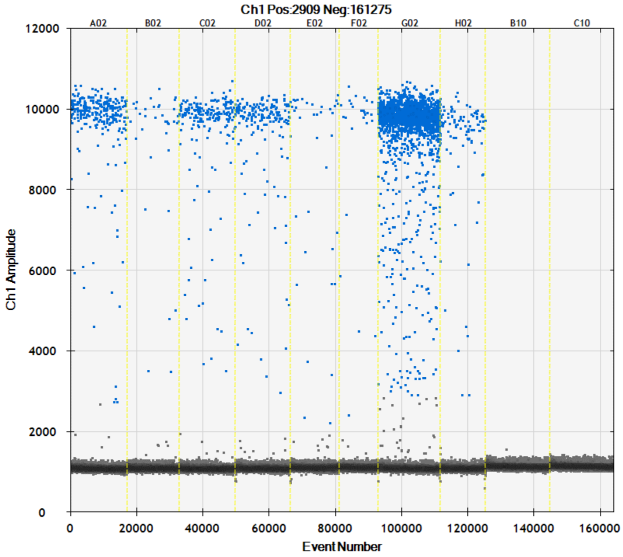	17352	9.6
miR-103a-3p	000439	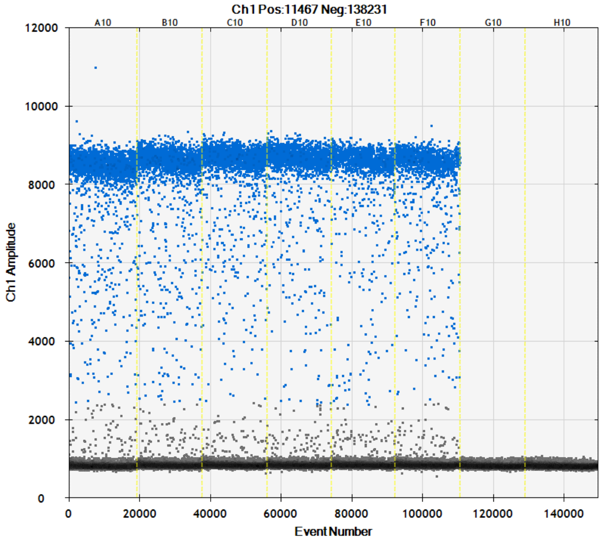	16713	10.0
miR-203a-3p	000507	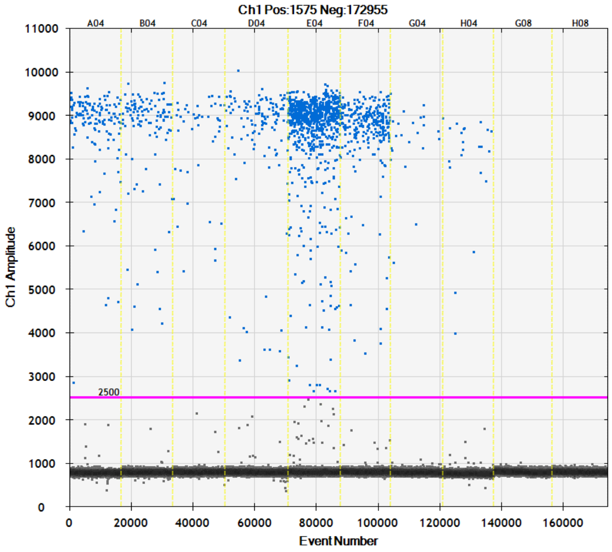	17434	7.5

**Gene name miRBase v.22**	**AB assay ID**	**Fluorescence amplitudes of positive and negative droplets/assay**	**Mean number of accepted droplets/well**	**%R SD**
miR-204-5p	000508	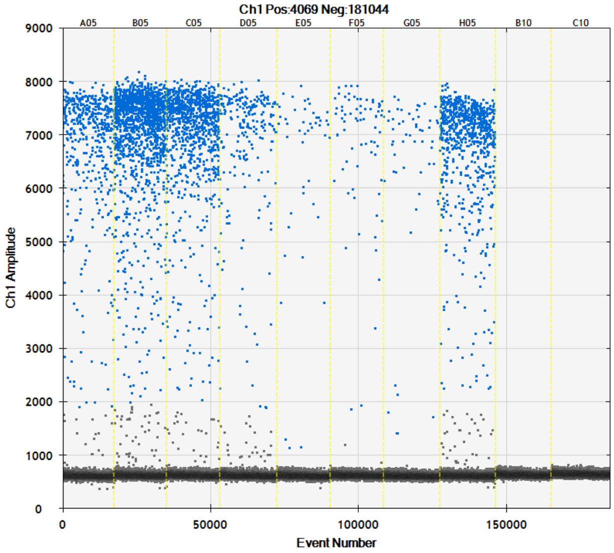	18000	7.5
miR-223-3p	002295	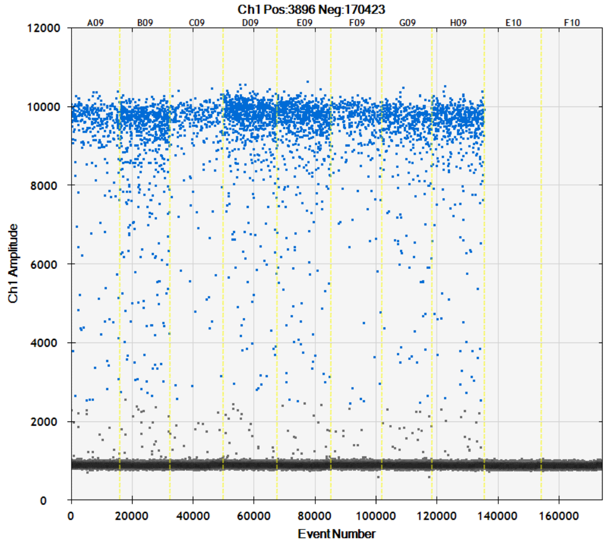	17314	9.3
miR-489-3p	002358	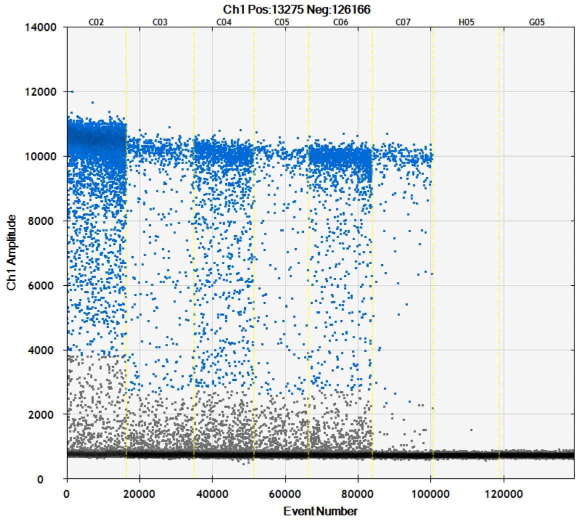	17736	7.3

Gene name miRBase v.22	AB assay ID	Fluorescence amplitudes of positive and negative droplets/assay	Mean number of accepted droplets/well	%RSD
miR-500a-5p	002428	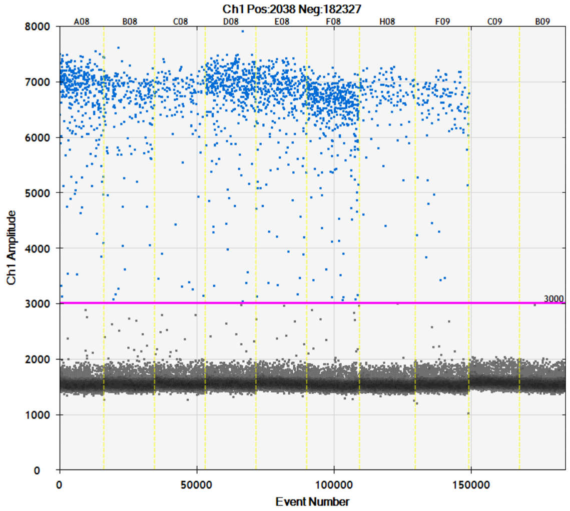	16963	10.4
miR-885-5p	002296	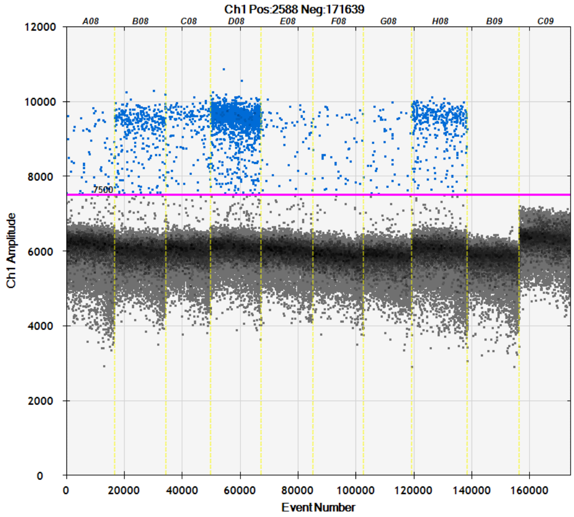	17151	8.0

Figure S3. Fluorescence scatter plots of the droplet digital PCR miRNA assays The blue clusters represent positive droplets with successful PCR amplification and the black clusters represent negative droplets without amplification. The threshold between positive and negative droplets can be set either automatically by the QuantaSoft software or manually (corresponding to the red line in the plot of miR-203a-3p, miR-500a-5p, and miR-885-5p). Outlier wells were indicated by the software with to many positive droplets, with quality score below 0.85 (corresponding with the drop volume of <0.85 nL) or wells with fewer than 10 000 droplets. The mean number of accepted droplets and %RSD was calculated using all measurements in this study. Abbreviation: %RSD = percent relative standard deviation.

Table S7. Parameter λ given as median copies and interquartile range per partition for the measured miRNAs. λ was calculated as ratio of the estimated copy number in the total volume in all partitions (m) to the number of partitions (n).

λ statistics per miR	miR-9-5p	miR-103-3p	miR-203-3p	miR-204-5p	miR-223-3p	miR-489-3p	miR-500-5p	miR-885-5p
**Median**	0.0112	0.139	0.0055	0.0221	0.0284	0.0305	0.0153	0.0068

**IQR***	0.0039 to 0.0371	0.0754 to 0.2020	0.00215 to 0.0140	0.0035 to 0.1063	0.0134 to 0.0546	0.0194 to 0.0775	0.0072 to 0.0291	0.0042 to 0.0209

*IQR = interquartile range.

Performance data of the droplet digital PCR analyses

Repeatability and reproducibility of measurements are presented as analytical performance data in the following Supplementary Table S8.

Table S8. Repeatability and reproducibility data of ddPCR analyses. Repeatability data (**A**) were calculated as percent RSD values based on duplicate measurements of miR-103a-3p in this study. Reproducibility data (**B**) were calculated both for the copies of all miRNAs and the accepted droplets in their analyses using different levels of control materials.

A. Repeatability data.

**miR-103a-3p**	**Copy Number/µL**	**Accepted Droplets/Well**
Sample size	126	126
Overall mean	132.8	16662
SD	6.57	798
**%RSD**	**4.95**	**4.79**

B. Reproducibility data.

**Hsa-miR**	**Copy Number/µL**		**Accepted Droplets/well**		**Replicates**
	Mean (SD)	%RSD	Mean (SD)	%RSD	(n)
miR-9-5p					
level 1	1.7 (0.19)	11.3	17271 (1545)	8.9	4
level 2	15.2 (1.09)	7.2	18104 (896)	4.9	5
level 3	145.2 (6.42)	4.4	17663 (2454)	13.9	5
miR-103a-3p					
level 1	96.6 (6.39)	6.6	17573 (955)	5.4	8
miR-203a-3p					
level 1	3.6 (0.44)	12.1	18786 (903)	4.8	6
level 2	13.4 (1.23)	9.2	18106 (589)	3.3	8
miR-204-5p					
level 1	2.6 (0.40)	15.4	18002 (645)	3.6	6
level 2	26.4 (2.61)	9.9	17396 (1346)	7.7	6
miR-223-3p					
level 1	22.5 (1.76)	7.8	17334 (308)	1.8	4
level 2	35.4 (2.99)	8.4	17827 (1396)	7.8	5
miR-489-3p					
level 1	24.2 (1.15)	4.8	17386 (1328)	7.6	5
level 2	106.0 (3.39)	3.2	17866 (294)	1.6	5
miR-500a-5p					
level 1	7.2 (0.58)	8.0	17603 (1023)	5.8	6
level 2	24.4 (1.40)	5.7	19083 (1035)	5.4	5
miR-885-5p					
level 1	3.4 (0.51)	15.0	16916 (1131)	6.7	4
level 2	22.0 (2.10)	9.6	18437 (1057)	5.7	5

Additional expression and correlation data

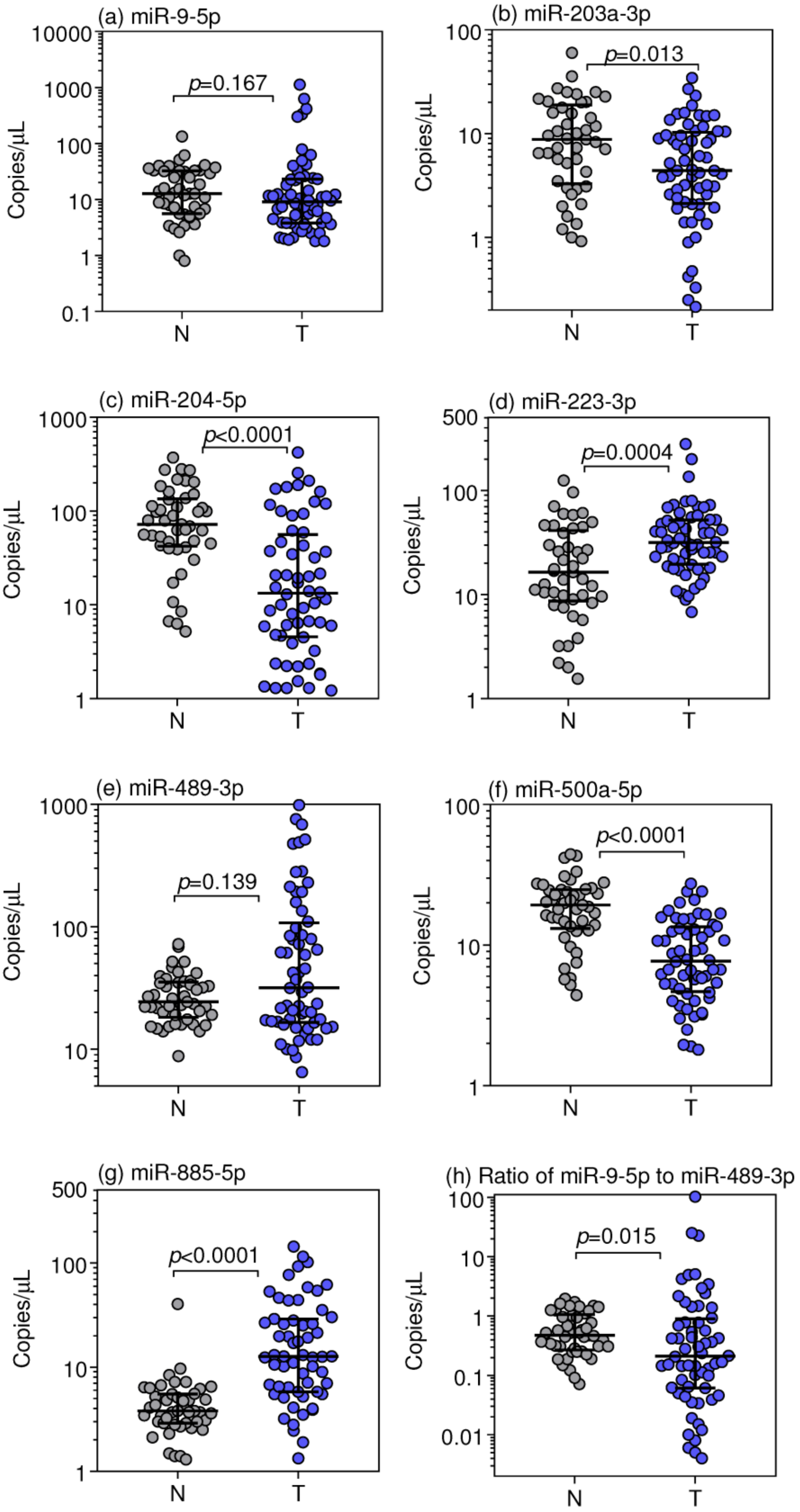

Figure S4. Expression of selected miRNAs in tumor samples of RCC patients compared to normal adjacent renal parenchyma. Values were measured using droplet digital RT-PCR and are represented as medians with interquartile ranges of 60 tumor samples (T) and 45 samples from normal renal parenchyma distant from tumor (N). Significant differences between the sample groups were calculated by Mann-Whitney *U* test.

Table S9. Spearman rank correlations between pathological variables and miRNAs. Data were calculated with the total cohort as the correlation coefficients did not differ (*p* <0.100) between the responder and non-responder patients.

Correlation between pathological variables and miRNAs		miR-9-p	miR-203a-3p	miR-204-5p	miR-223-3p	miR-489-3p	miR-500a-5p	miR-885-5p
pT stage	Spearman R_S	0.092	-0.066	-0.183	0.103	0.080	-0.127	-0.075
	p value	0.483	0.617	0.162	0.434	0.543	0.332	0.571
Fuhrman grade	Spearman R_S	**0.324**	0.008	**-0.311**	-0.179	-0.033	-0.125	-0.168
	p value	**0.013**	0.952	**0.017**	0.178	0.808	0.348	0.207
Metastatic status	Spearman R_S	0.152	-0.046	0.015	0.012	-0.043	-0.109	-0.177
	p value	0.245	0.725	0.907	0.930	0.742	0.407	0.177

Table S10. List of the experimentally validated target genes of miR-9-5p taken from the databases miRTarBase [7], miRWalk2.0 [8], and Diana-Tarbase v.7.0 [9].

**Gene**	**EntrezID**	**Chr**	**Map**	**Definition**
ABCC1	4363	16	16p13.1	ATP-binding cassette sub-family C CFTR/MRP member 1
ACAA2	10449	18	18q21.1	acetyl-CoA acyltransferase 2
ACADSB	36	10	10q26.13	acyl-CoA dehydrogenase short/branched chain
ACAT1	38	11	11q22.3	acetyl-CoA acetyltransferase 1
AGAP9	642517	10	10q11.22	ArfGAP with GTPase domain ankyrin repeat and PH domain 9
AKR1B10	57016	7	7q33	aldo-keto reductase family 1 member B10 aldose reductase
AKR1C8P	340811	10	10p15.1	aldo-keto reductase family 1, member C8, pseudogene
ALDH1A3	220	15	15q26.3	aldehyde dehydrogenase 1 family member A3
AMOTL1	154810	11	11q14.3	angiomotin like 1
ANKFY1	51479	17	17p13.3	ankyrin repeat and FYVE domain containing 1
ANP32B	10541	9	9q22.32	acidic leucine-rich nuclear phosphoprotein 32 family member B
AP3B1	8546	5	5q14.1	adaptor-related protein complex 3 beta 1 subunit
APBB2	323	4	4p13	amyloid beta A4 precursor protein-binding family B member 2
ARHGEF10	9639	8	8p23	Rho guanine nucleotide exchange factor GEF 10
ARL8B	55207	3	3p26.1	ADP-ribosylation factor-like 8B
ATG10	83734	5	5q14.1	autophagy related 10
ATL1	51062	14	14q22.1	atlastin GTPase 1
ATP11C	286410	X	Xq27.1	ATPase class VI type 11C
ATP6V1C2	245973	2	NA	ATPase H+ transporting lysosomal 42kDa V1 subunit C2
ATP7A	538	X	Xq21.1	ATPase Cu++ transporting alpha polypeptide
ATXN7	6314	3	3p21.1-p12	ataxin 7
AUH	549	9	9q22.31	AU RNA binding protein/enoyl-CoA hydratase
AVIL	10677	12	12q14.1	advillin
BACE1	23621	11	11q23.2-q23.3	beta-site APP-cleaving enzyme 1
BCL2L11	10018	2	2q13	BCL2-like 11 apoptosis facilitator
BCL6	604	3	3q27	B-cell CLL/lymphoma 6
BIK	638	22	22q13.31	BCL2-interacting killer apoptosis-inducing
BOD1L1	259282	4	4p16.1	biorientation of chromosomes in cell division 1-like 1
C7orf31	136895	7	7p15.3	chromosome 7 open reading frame 31
CA13	377677	8	8q21.2	carbonic anhydrase XIII
CA8	767	8	8q12.1	carbonic anhydrase VIII
CALML5	51806	10	10p15.1	calmodulin-like 5
CAPZA1	829	1	1p13.2	capping protein actin filament muscle Z-line alpha 1
CARD19	84270	9	9q22.31	caspase recruitment domain family member 19
CCDC152	100129792	5	5p12	coiled-coil domain containing 152
CCL19	6363	9	9p13	chemokine C-C motif ligand 19
CCND1	595	11	11q13	cyclin D1
CCNDBP1	23582	15	15q14-q15	cyclin D-type binding-protein 1

Gene	EntrezID	Chr	Map	Definition
CCNG1	900	5	5q32-q34	cyclin G1
CD34	947	1	1q32	CD34 molecule
CD46	4179	1	1q32	CD46 molecule complement regulatory protein
CDH1	999	16	16q22.1	cadherin 1 type 1 E-cadherin epithelial
CDH3	1001	16	16q22.1	cadherin 3 type 1 P-cadherin placental
CDH7	1005	18	18q22.1	cadherin 7 type 2
CDK12	51755	17	17q12	cyclin-dependent kinase 12
CDX2	1045	13	13q12.3	caudal type homeobox 2
CERS2	29956	1	1q21.3	ceramide synthase 2
CERS4	79603	19	19p13.2	ceramide synthase 4
CHMP2B	25978	3	3p11.2	charged multivesicular body protein 2B
CHMP3	51652	2	2p11.2	charged multivesicular body protein 3
CHSY1	22856	15	15q26.3	chondroitin sulfate synthase 1
CMKLR1	1240	12	12q24.1	chemokine-like receptor 1
CMTM2	146225	16	16q21	CKLF-like MARVEL transmembrane domain containing 2
CNOT6	57472	5	5q35.3	CCR4-NOT transcription complex subunit 6
COL12A1	1303	6	6q12-q13	collagen type XII alpha 1
COL6A5	256076	3	3q22.1	collagen type VI alpha 5
COLEC12	81035	18	18p11.32	collectin sub-family member 12
CPA4	51200	7	7q32	carboxypeptidase A4
CPEB4	80315	5	5q21	cytoplasmic polyadenylation element binding protein 4
CREB1	1385	2	2q34	cAMP responsive element binding protein 1
CTHRC1	115908	8	8q22.3	collagen triple helix repeat containing 1
CUEDC1	404093	17	17q23.2	CUE domain containing 1
CXCR4	7852	2	2q21	chemokine C-X-C motif receptor 4
CXXC5	51523	5	5q31.2	CXXC finger protein 5
CYFIP2	26999	5	5q33.3	cytoplasmic FMR1 interacting protein 2
CYP39A1	51302	6	6p21.1-p11.2	cytochrome P450 family 39 subfamily A polypeptide 1
DICER1	23405	14	14q32.13	dicer 1 ribonuclease type III
DMXL1	1657	5	5q22	Dmx-like 1
DRD2	1813	11	11q23	dopamine receptor D2
DSP	1832	6	6p24	desmoplakin
E2F7	144455	12	12q21.2	E2F transcription factor 7
EDEM3	80267	1	1q25	ER degradation enhancer mannosidase alpha-like 3
EEF2K	29904	16	16p12.2	eukaryotic elongation factor-2 kinase
EFCAB14	9813	1	1p33	EF-hand calcium binding domain 14
EFNA1	1942	1	1q21-q22	ephrin-A1
ELAVL1	1994	19	19p13.2	ELAV like RNA binding protein 1
ELF3	1999	1	1q32.2	E74-like factor 3 ets domain transcription factor epithelial-specific
EMC1	23065	1	1p36.13	ER membrane protein complex subunit 1
EN2	2020	7	7q36	engrailed homeobox 2
ENDOD1	23052	11	11q21	endonuclease domain containing 1
EOGT	285203	3	3p14.1	EGF domain-specific O-linked N-acetylglucosamine GlcNAc transferase
EP300	2033	22	22q13.2	E1A binding protein p300
ESR1	2099	6	6q25.1	estrogen receptor 1
ETS1	2113	11	11q23.3	v-ets avian erythroblastosis virus E26 oncogene homolog 1
F2	2147	11	11p11	coagulation factor II thrombin
FAIM	55179	3	3q22.3	Fas apoptotic inhibitory molecule
FAM46A	55603	6	6q14	family with sequence similarity 46 member A
FAM73B	84895	9	9q34.11	family with sequence similarity 73 member B
FBN2	2201	5	5q23-q31	fibrillin 2
FERMT1	55612	20	20p12.3	fermitin family member 1

Gene	EntrezID	Chr	Map	Definition
FERMT2	10979	14	14q22.1	fermitin family member 2
FLNB	2317	3	3p14.3	filamin B beta
FNBP1	23048	9	9q34	formin binding protein 1
FOXO1	2308	13	13q14.1	forkhead box O1
FOXO3	2309	6	6q21	forkhead box O3
FOXP1	27086	3	3p14.1	forkhead box P1
FRMD4A	55691	10	10p13	FERM domain containing 4A
FRMD4B	23150	3	3p14.1	FERM domain containing 4B
FSTL3	10272	19	19p13	follistatin-like 3 secreted glycoprotein
GALNTL6	442117	4	4q34.1	polypeptide N-acetylgalactosaminyltransferase-like 6
GFOD1	54438	6	6pter-p22.1	glucose-fructose oxidoreductase domain containing 1
GIGYF1	64599	7	7q22	GRB10 interacting GYF protein 1
GIP	2695	17	17q21.3-q22	gastric inhibitory polypeptide
GNAT2	2780	1	1p13.1	guanine nucleotide binding protein G protein alpha transducing activity polypeptide 2
GPBP1L1	60313	1	1p34.1	GC-rich promoter binding protein 1-like 1
GRN	2896	17	17q21.32	granulin
HBP1	26959	7	7q22-q31	HMG-box transcription factor 1
HDAC4	9759	2	2q37.3	histone deacetylase 4
HIST1H2AE	3012	6	6p22.1	histone cluster 1 H2ae
HIST1H2AI	8329	6	6p22.1	histone cluster 1 H2ai
HIST1H3F	8968	6	6p22.2	histone cluster 1 H3f
HIST1H4H	8365	6	6p22.1	histone cluster 1 H4h
HIST2H2AC	8338	1	1q21.2	histone cluster 2 H2ac
HLA-A	3105	6	6p21.3	major histocompatibility complex class I A
HN1L	90861	16	16p13.3	hematological and neurological expressed 1-like
HOXC12	3228	12	12q13.13	homeobox C12
HTR3A	3359	11	11q23.1	5-hydroxytryptamine serotonin receptor 3A ionotropic
ID2	3398	2	2p25	inhibitor of DNA binding 2 dominant negative helix-loop-helix protein
ID4	3400	6	6p22.3	inhibitor of DNA binding 4 dominant negative helix-loop-helix protein
IDS	3423	X	Xq28	iduronate 2-sulfatase
IGF2R	3482	6	6q26	insulin-like growth factor 2 receptor
IL5	3567	5	5q31.1	interleukin 5
IPO7	10527	11	11p15.4	importin 7
IPPK	64768	9	9q22.31	inositol 13456-pentakisphosphate 2-kinase
IREB2	3658	15	15q25.1	iron-responsive element binding protein 2
ITGB3BP	23421	1	1p31.3	integrin beta 3 binding protein beta3-endonexin
JAK1	3716	1	1p32.3-p31.3	Janus kinase 1
KCNJ15	3772	21	21q22.2	potassium inwardly-rectifying channel subfamily J member 15
KCNJ2	3759	17	17q24.3	potassium inwardly-rectifying channel subfamily J member 2
KIAA1468	57614	18	18q21.33	KIAA1468
KIF1A	547	2	2q37.3	kinesin family member 1A
KIF1B	23095	1	1p36.2	kinesin family member 1B
KIF1C	10749	17	17p13.2	kinesin family member 1C
KLF17	128209	1	1p34.1	Kruppel-like factor 17
KLF5	688	13	13q22.1	Kruppel-like factor 5 intestinal
KLF6	1316	10	10p15	Kruppel-like factor 6
KLRC1	3821	12	12p13	killer cell lectin-like receptor subfamily C member 1
KLRK1	22914	12	12p13.2-p12.3	killer cell lectin-like receptor subfamily K member 1
KMT2D	8085	12	12q13.12	lysine K-specific methyltransferase 2D
LDLRAP1	26119	1	1p36-p35	low density lipoprotein receptor adaptor protein 1
LHFPL2	10184	5	5q14.1	lipoma HMGIC fusion partner-like 2
LMNA	4000	1	1q22	lamin A/C

Gene	EntrezID	Chr	Map	Definition
LPPR2	64748	19	19p13.2	lipid phosphate phosphatase-related protein type 2
LPXN	9404	11	11q12.1	leupaxin
LRP1	4035	12	12q13.3	low density lipoprotein receptor-related protein 1
LRRC15	131578	3	3q29	leucine rich repeat containing 15
LRRTM1	347730	2	2p12	leucine rich repeat transmembrane neuronal 1
MAGEA2B	266740	X	Xq28	melanoma antigen family A 2B
MALAT1	378938	11	11q13.1	metastasis associated lung adenocarcinoma transcript 1 (non-protein coding)
MAP1B	4131	5	5q13	microtubule-associated protein 1B
MAP3K3	4215	17	17q23.3	mitogen-activated protein kinase kinase kinase 3
MAP3K8	1326	10	10p11.23	mitogen-activated protein kinase kinase kinase 8
MCC	4163	5	5q21	mutated in colorectal cancers
MDM4	4194	1	1q32	MDM4 p53 regulator
MESDC1	59274	15	15q13	mesoderm development candidate 1
MKX	283078	10	10p12.1	mohawk homeobox
MMP13	4322	11	11q22.3	matrix metallopeptidase 13 collagenase 3
MMP2	4313	16	16q13-q21	matrix metallopeptidase 2 gelatinase A 72kDa gelatinase 72kDa type IV collagenase
MMP9	4318	20	20q11.2-q13.1	matrix metallopeptidase 9 gelatinase B 92kDa gelatinase 92kDa type IV collagenase
MN1	4330	22	22q12.1	meningioma disrupted in balanced translocation 1
MORF4L1	10933	15	15q24	mortality factor 4 like 1
MSR1	4481	8	8p22	macrophage scavenger receptor 1
MTHFD1	4522	14	14q24	methylenetetrahydrofolate dehydrogenase NADP+ dependent 1 methenyltetrahydrofolate cyclohydrolase formyltetrahydrofolate synthetase
MTHFD2	10797	2	2p13.1	methylenetetrahydrofolate dehydrogenase NADP+ dependent 2 methenyltetrahydrofolate cyclohydrolase
MTL5	9633	11	11q13.2-q13.3	metallothionein-like 5 testis-specific tesmin
MTX3	345778	5	5q14.1	metaxin 3
MYF6	4618	12	12q21	myogenic factor 6 herculin
MYH9	4627	22	22q13.1	myosin heavy chain 9 non-muscle
MYLK	4638	3	3q21	myosin light chain kinase
NAV1	89796	1	1q32.3	neuron navigator 1
NBEA	26960	13	13q13	neurobeachin
NCOR2	9612	12	12q24	nuclear receptor corepressor 2
NF1	4763	17	17q11.2	neurofibromin 1
NFAT5	10725	16	16q22.1	nuclear factor of activated T-cells 5 tonicity-responsive
NFATC3	4775	16	16q22.2	nuclear factor of activated T-cells cytoplasmic calcineurin-dependent 3
NFKB1	4790	4	4q24	nuclear factor of kappa light polypeptide gene enhancer in B-cells 1
NIN	51199	14	14q22.1	ninein GSK3B interacting protein
NIPA1	123606	15	15q11.2	non imprinted in Prader-Willi/Angelman syndrome 1
NKX2-2	4821	20	20p11.22	NK2 homeobox 2
NMNAT2	23057	1	1q25	nicotinamide nucleotide adenylyltransferase 2
NPY4R	5540	10	10q11.2	neuropeptide Y receptor Y4
NR2E1	7101	6	6q21	nuclear receptor subfamily 2 group E member 1
NR6A1	2649	9	9q33.3	nuclear receptor subfamily 6 group A member 1
NRIP3	56675	11	11p15.3	nuclear receptor interacting protein 3
NSUN7	79730	4	4p14	NOP2/Sun domain family member 7
NTRK3	4916	15	15q25	neurotrophic tyrosine kinase receptor type 3
NUDT19	390916	19	19q13.11	nudix nucleoside diphosphate linked moiety X-type motif 19
NUP155	9631	5	5p13.1	nucleoporin 155kDa
OAF	220323	11	11q23.3	OAF homolog Drosophila
OLR1	4973	12	12p13.2-p12.3	oxidized low density lipoprotein lectin-like receptor 1

Gene	EntrezID	Chr	Map	Definition
ONECUT2	9480	18	18q21.31	one cut homeobox 2
OPTN	10133	10	10p13	optineurin
P4HA2	8974	5	5q31	prolyl 4-hydroxylase alpha polypeptide II
PACS1	55690	11	11q13.1-q13.2	phosphofurin acidic cluster sorting protein 1
PAX1	5075	20	20p11.2	paired box 1
PCMTD2	55251	20	20q13.33	protein-L-isoaspartate D-aspartate O-methyltransferase domain containing 2
PDZK1	5174	1	1q21	PDZ domain containing 1
PELP1	27043	17	17p13.2	proline glutamate and leucine rich protein 1
PI4K2A	55361	10	10q24	phosphatidylinositol 4-kinase type 2 alpha
PIGB	9488	15	15q21.3	phosphatidylinositol glycan anchor biosynthesis class B
PIGM	93183	1	1q23.2	phosphatidylinositol glycan anchor biosynthesis class M
PLCB4	5332	20	20p12	phospholipase C beta 4
PLEKHB1	58473	11	11q13.5-q14.1	pleckstrin homology domain containing family B evectins member 1
PNRC2	55629	1	1p36.11	proline-rich nuclear receptor coactivator 2
POGZ	23126	1	1q21.3	pogo transposable element with ZNF domain
POU2F1	5451	1	1q24.2	POU class 2 homeobox 1
POU2F2	5452	19	19q13.2	POU class 2 homeobox 2
PPAP2A	8611	5	5q11	phosphatidic acid phosphatase type 2A
PPARA	5465	22	22q13.31	peroxisome proliferator-activated receptor alpha
PPP4R2	151987	3	3p13	protein phosphatase 4 regulatory subunit 2
PQLC3	130814	2	2p25.1	PQ loop repeat containing 3
PRDM1	639	6	6q21	PR domain containing 1 with ZNF domain
PRG4	10216	1	1q25-q31	proteoglycan 4
PRKD3	23683	2	2p21	protein kinase D3
PRRX1	5396	1	1q24	paired related homeobox 1
PRTG	283659	15	15q21.3	protogenin
PTPRK	5796	6	6q22.2-q22.3	protein tyrosine phosphatase receptor type K
PXDN	7837	2	2p25	peroxidasin homolog Drosophila
QARS	5859	3	3p21.31	glutaminyl-tRNA synthetase
RAB34	83871	17	17q11.2	RAB34 member RAS oncogene family
RAB8B	51762	15	15q22.2	RAB8B member RAS oncogene family
RABGAP1	23637	9	9q34.11	RAB GTPase activating protein 1
RASSF8	11228	12	12p12.3	Ras association RalGDS/AF-6 domain family N-terminal member 8
RBFOX2	23543	22	22q13.1	RNA binding protein fox-1 homolog C. elegans 2
RCC2	55920	1	1p36.13	regulator of chromosome condensation 2
REST	5978	4	4q12	RE1-silencing transcription factor
RHAG	6005	6	6p12.3	Rh-associated glycoprotein
RHOH	399	4	4p13	ras homolog family member H
RHOV	171177	15	15q13.3	ras homolog family member V
RNF103-CHMP3	100526767	2	2p	RNF103-CHMP3 readthrough
RNF146	81847	6	6q22.1-q22.33	ring finger protein 146
RNF44	22838	5	5q35.2	ring finger protein 44
RYBP	23429	3	3p13	RING1 and YY1 binding protein
SCD5	79966	4	4q21.22	stearoyl-CoA desaturase 5
SDC1	6382	2	2p24.1	syndecan 1
SEC23IP	11196	10	10q25-q26	SEC23 interacting protein
SERAC1	84947	6	6q25.3	serine active site containing 1
SERINC5	256987	5	5q14.1	serine incorporator 5
SERPINB9	5272	6	6p25	serpin peptidase inhibitor clade B ovalbumin member 9
SERPINH1	871	11	11q13.5	serpin peptidase inhibitor clade H heat shock protein 47 member 1 collagen binding protein 1

Gene	EntrezID	Chr	Map	Definition
SFT2D2	375035	1	1q24.2	SFT2 domain containing 2
SIRT1	23411	10	10q21.3	sirtuin 1
SLC19A3	80704	2	2q37	solute carrier family 19 thiamine transporter member 3
SLC22A3	6581	6	6q25.3	solute carrier family 22 organic cation transporter member 3
SLC22A5	6584	5	5q23.3	solute carrier family 22 organic cation/carnitine transporter member 5
SLC25A30	253512	13	13q14.13	solute carrier family 25 member 30
SLC2A5	6518	1	1p36.2	solute carrier family 2 facilitated glucose/fructose transporter member 5
SLC30A4	7782	15	15q21.1|15q21.1	solute carrier family 30 zinc transporter member 4
SLC35B3	51000	6	6p24.3	solute carrier family 35 adenosine 3'-phospho 5'-phosphosulfate transporter member B3
SLC35E2	9906	1	1p36.33	solute carrier family 35 member E2
SLC39A14	23516	8	8p21.3	solute carrier family 39 zinc transporter member 14
SLC7A2	6542	8	8p22	solute carrier family 7 cationic amino acid transporter y+ system member 2
SMARCA1	6594	X	Xq25	SWI/SNF related matrix associated actin dependent regulator of chromatin subfamily a member 1
SMU1	55234	9	9p12	smu-1 suppressor of mec-8 and unc-52 homolog C. elegans
SNAI2	6591	8	8q11	snail family zinc finger 2
SNX7	51375	1	1p21.3	sorting nexin 7
SOCS5	9655	2	2p21	suppressor of cytokine signaling 5
SOD2	6648	6	6q25.3	superoxide dismutase 2 mitochondrial
SPAG9	9043	17	17q21.33	sperm associated antigen 9
SPI1	6688	11	11p11.2	spleen focus forming virus SFFV proviral integration oncogene
SPON2	10417	4	4p16.3	spondin 2 extracellular matrix protein
SPTBN1	6711	2	2p21	spectrin beta non-erythrocytic 1
SRF	6722	6	6p21.1	serum response factor c-fos serum response element-binding transcription factor
STK3	6788	8	8q22.2	serine/threonine kinase 3
STMN1	3925	1	1p36.11	stathmin 1
STX6	10228	1	1q25.3	syntaxin 6
SUMF2	25870	7	7q11.1	sulfatase modifying factor 2
SUMO1	7341	2	2q33	small ubiquitin-like modifier 1
SYAP1	94056	X	Xp22.2	synapse associated protein 1
SYNE1	23345	6	6q25	spectrin repeat containing nuclear envelope 1
SYNE2	23224	14	14q23.2	spectrin repeat containing nuclear envelope 2
SYNGR2	9144	17	17q25.3	synaptogyrin 2
SYPL1	6856	7	7q22.3	synaptophysin-like 1
TAGLN	6876	11	11q23.2	transgelin
TAL1	6886	1	1p32	T-cell acute lymphocytic leukemia 1
TAOK1	57551	17	17q11.2	TAO kinase 1
TBC1D9	23158	4	4q31.21	TBC1 domain family member 9 with GRAM domain
TBPL1	9519	6	6q22.1-q22.3	TBP-like 1
TC2N	123036	14	14q32.12	tandem C2 domains nuclear
TCL1B	9623	14	14q32.1	T-cell leukemia/lymphoma 1B
TESK2	10420	1	1p32	testis-specific kinase 2
TFRC	7037	3	3q29	transferrin receptor
TGFBI	7045	5	5q31	transforming growth factor beta-induced 68kDa
TGFBR2	7048	3	3p22	transforming growth factor beta receptor II 70/80kDa
TMEM216	51259	11	11q13.1	transmembrane protein 216
TMEM245	23731	9	9q31	transmembrane protein 245
TRAK2	66008	2	2q33	trafficking protein kinesin binding 2
TRIM14	9830	9	9q22.33	tripartite motif containing 14
TRMT13	54482	1	1p21.2	tRNA methyltransferase 13 homolog *S. cerevisiae*

Gene	EntrezID	Chr	Map	Definition
TRPM7	54822	15	15q21	transient receptor potential cation channel subfamily M member 7
TRRAP	8295	7	7q21.2-q22.1	transformation/transcription domain-associated protein
TTC7B	145567	14	14q32.11	tetratricopeptide repeat domain 7B
TWISTNB	221830	7	7p21.1	TWIST neighbor
UBE4B	10277	1	1p36.3	ubiquitination factor E4B
UGDH	7358	4	4p15.1	UDP-glucose 6-dehydrogenase
UHMK1	127933	1	1q23.3	U2AF homology motif UHM kinase 1
UMPS	7372	3	3q13	uridine monophosphate synthetase
VAMP5	10791	2	2p11.2	vesicle-associated membrane protein 5
VEGFA	7422	6	6p12	vascular endothelial growth factor A
VIM	7431	10	10p13	vimentin
VPS8	23355	3	3q27.2	vacuolar protein sorting 8 homolog *S. cerevisiae*
WDR70	55100	5	5p13.2	WD repeat domain 70
WNT6	7475	2	2q35	wingless-type MMTV integration site family member 6
WNT8A	7478	5	5q31	wingless-type MMTV integration site family member 8A
XRN2	22803	20	20p11.2-p11.1	5'-3' exoribonuclease 2
ZBED3	84327	5	5q13.3	zinc finger BED-type containing 3
ZFAND1	79752	8	8q21.13	zinc finger AN1-type domain 1
ZMAT4	79698	8	8p11.21	zinc finger matrin-type 4
ZNF407	55628	18	18q23	zinc finger protein 407
ZNF557	79230	19	19p13.2	zinc finger protein 557

References in the supplementary materials

1. Bustin, S.A.; Benes, V.; Garson, J.A.; Hellemans, J.; Huggett, J.; Kubista, M.; Mueller, R.; Nolan, T.; Pfaffl, M.W.; Shipley, G.L.; et al. The MIQE guidelines: Minimum information for publication of quantitative real-time PCR experiments. *Clin. Chem.* **2009**, *55*, 611–622.
2. Huggett, J.F.; Foy, C.A.; Benes, V.; Emslie, K.; Garson, J.A.; Haynes, R.; Hellemans, J.; Kubista, M.; Mueller, R.D.; Nolan, T.; et al. The digital MIQE guidelines: Minimum Information for Publication of Quantitative Digital PCR Experiments. *Clin. Chem.* **2013**, *59*, 892–902.
3. Jung, M.; Schaefer, A.; Steiner, I.; Kempkensteffen, C.; Stephan, C.; Erbersdobler, A.; Jung, K. Robust microRNA stability in degraded RNA preparations from human tissue and cell samples. *Clin. Chem.* **2010**, *56*, 998–1006.
4. Jung, M.; Mollenkopf, H.J.; Grimm, C.; Wagner, I.; Albrecht, M.; Waller, T.; Pilarsky, C.; Johannsen, M.; Stephan, C.; Lehrach, H.; et al. MicroRNA profiling of clear cell renal cell cancer identifies a robust signature to define renal malignancy. *J. Cell. Mol. Med.* **2009**, *13*, 3918–3928.
5. Schaefer, A.; Jung, M.; Mollenkopf, H.J.; Wagner, I.; Stephan, C.; Jentzmik, F.; Miller, K.; Lein, M.; Kristiansen, G.; Jung, K. Diagnostic and prognostic implications of microRNA profiling in prostate carcinoma. *Int. J. Cancer* **2010**, *126*, 1166–1176.
6. Wotschofsky, Z.; Meyer, H.A.; Jung, M.; Fendler, A.; Wagner, I.; Stephan, C.; Busch, J.; Erbersdobler, A.; Disch, A.C.; Mollenkopf, H.J.; et al. Reference genes for the relative quantification of microRNAs in renal cell carcinomas and their metastases. *Anal. Biochem.* **2011**, *417*, 233–241.
7. Chou, C.H.; Chang, N.W.; Shrestha, S.; Hsu, S.D.; Lin, Y.L.; Lee, W.H.; Yang, C.D.; Hong, H.C.; Wei, T.Y.; Tu, S.J.; et al. miRTarBase 2016: Updates to the experimentally validated miRNA-target interactions database. *Nucleic Acids Res.* **2016,** *44*, D239-D247.
8. Dweep, H.; Gretz, N. miRWalk2.0: A comprehensive atlas of microRNA-target interactions. *Nat. Methods* **2015**, *12*, 697. doi: 10.1038/nmeth.3485.
9. Vlachos, I.S.; Paraskevopoulou, M.D.; Karagkouni, D.; Georgakilas, G.; Vergoulis, T.; Kanellos, I.; Anastasopoulos, I.L.; Maniou, S.; Karathanou, K.; Kalfakakou, D.; et al. DIANA-TarBase v7.0: Indexing more than half a million experimentally supported miRNA:mRNA interactions. *Nucleic Acids Res.* **2015,** *43*, D153-D159.

© 2018 by the authors. Licensee MDPI, Basel, Switzerland. This article is an open access article distributed under the terms and conditions of the Creative Commons Attribution (CC BY) license (http://creativecommons.org/licenses/by/4.0/).
